# Photoregulatory functions drive variation in eye coloration across macaque species

**DOI:** 10.1038/s41598-024-80643-4

**Published:** 2024-11-24

**Authors:** Juan Olvido Perea-García, Jorg J. M. Massen, Julia Ostner, Oliver Schülke, Alba Castellano-Navarro, Eva Gazagne, Juan Manuel José-Domínguez, Víctor Beltrán-Francés, Stefano Kaburu, Nadine Ruppert, Jérôme Micheletta, Shreejata Gupta, Bonaventura Majolo, Laëtitia Maréchal, Lena S. Pflüger, Pia M. Böhm, Marie Bourjade, Elif Duran, Catherine Hobaiter, Antónia Monteiro

**Affiliations:** 1 Department of Biological Sciences, National University of Singapore, Singapore, Singapore; 2 Center for Language Evolution Studies, Nicolaus Copernicus University, Toruń, Poland; 3 Animal Behaviour and Cognition, Department of Biology, Utrecht University, Utrecht, The Netherlands; 4 Department Behavioral Ecology, JFB Institute for Zoology and Anthropology, Georg-August University, Kellnerweg 6, 37077 Göttingen, Goettingen, Germany; 5 Research Group Primate Social Evolution, German Primate Center Leibniz Institute for Primate Research, Goettingen, Germany; 6 Leibniz Science Campus Primate Cognition, Goettingen, Germany; 7 Ethology and Animal Welfare Section, Universidad Cardenal Herrera-CEU, CEU Universities, Tirant lo Blanc 8, Alfara del Patriarca, Valencia, 46115 Spain; 8 Institute of Biology, Leipzig University, Leipzig, Germany; 9 Unit of Research SPHERES, University of Liège, Liège, Belgium; 10 Conservation Ecology Program, King Mongkut’s University of Technology, Bangkhuntien, Thailand; 11 Physical Anthropology Laboratory, Department of Legal Medicine, Toxicology and Physical Anthropology, University of Granada, Granada, Spain; 12 Fundació Universitat de Girona, Innovació I Formació, Girona, Spain; 13 School of Animal, Rural and Environmental Sciences, Nottingham Trent University, Nottingham, NG25 0QF UK; 14 School of Biological Sciences, Universiti Sains Malaysia, Gelugor, 11800 Pulau Pinang Malaysia; 15 Malaysian Primatological Society, Kulim, 09000 Kedah Malaysia; 16 Department of Psychology, Centre for Comparative and Evolutionary Psychology, University of Portsmouth, Portsmouth, UK; 17 Laboratoire de Psychologie Cognitive (LPC), Laboratoire Parole et Langage (LPL), CNRS, Aix-Marseille Université, Marseille, France; 18 School of Psychology, University of Lincoln, Lincoln, UK; 19 Department of Behavioral and Cognitive Biology, University of Vienna, Djerassiplatz 1, Vienna, 1030 Austria; 20 Wildlife Research Center, Kyoto University, 41-2 Kanrin Aichi, Inuyama, 484-8506 Japan; 21 CLLE, Université de Toulouse, CNRS, Toulouse, France; 22 Uaso Ngiro Baboon Project, Nairobi, Kenya; 23 Department of Psychology, Izmir University of Economics, Izmir, Turkey; 24 School of Psychology, University of St Andrews, Fife, Scotland, UK; 25 Science division, Yale-NUS College, Singapore, Singapore

**Keywords:** Coloration, Gloger’s rule, Ecology, Eyes, Light, Macaques, Photo-regulation, Primates, Ecology, Evolution, Zoology

## Abstract

Primates, the most colorful mammalian radiation, have previously served as an interesting model to test the functions and evolutionary drivers of variation in eye color. We assess the contribution of photo-regulatory and communicative functions to the external eye appearance of nine macaque species representing all the branches of their radiation. Macaques’ well described social structure and wide geographical distribution make them interesting to explore. We find that (1) the posterior option of the anterior eyeball is more pigmented closer to the equator, suggesting photoprotective functions. We also find that (2) the temporal side of the eyeball is more heavily pigmented than the nasal side. This suggests that eyeball pigmentation in macaques is distributed to reduce damage to the corneal limbus. The inclusion of a translocated population of *M. fuscata* in our analyses also suggests that external eye appearance may change quickly, perhaps owing to phenotypic plasticity. We find no evidence that communicative functions drive variation in external eye appearance in macaques. These results suggest that the amount of light in a species’ environment drives variation in eye coloration across macaque species. Furthermore, the geographical distribution of macaques hints at important factors that have yet to be accounted for, such as the reflectivity of the terrain a given species inhabits.

**Supplementary Information:**

The online version contains supplementary material available at 10.1038/s41598-024-80643-4.

## Introduction

Most hypotheses propose that primate eye color patterns function in intra- or inter-specific communication, rather than being shaped by photopic factors^1–7^. Surprisingly, given the vast variation of conditions of luminosity to which different primate species are exposed^8^ and the importance of vision in the taxon^9^, few studies examined the proposal that eye color is primarily serving a photo-regulatory function^10,11^. Kobayashi & Kohshima^1^ briefly considered photo-regulation as a driver of variation in conjunctivo-scleral pigmentation in primates, but they dismissed this possibility without quantitative examinations. Asymmetric ocular coloration patterns of macaques suggested photoprotective functions^10^. Perea-García et al.^11^. later supported this hypothesis by showing that conjunctival pigmentation diminishes with distance from the equator, similar to skin pigmentation in humans^12^. Their results showed that irises reflect more blue light with increasing distance from the equator, presumably due to a reduction in the number and size of melanosomes^13^. Even though more evidence is necessary to lend support to this finding^11^, ’s results suggested that iridal coloration made up for lack of blue light in septentrional latitudes, which is important for circadian rhythm regulation. Finally, developmental evidence^7,14,15^ and comparisons between sexes of the same species^14^ suggest that developmental constraints, and perhaps endocrinological differences, play a role in explaining patterns of color variation within species.

**Fig. 1:**
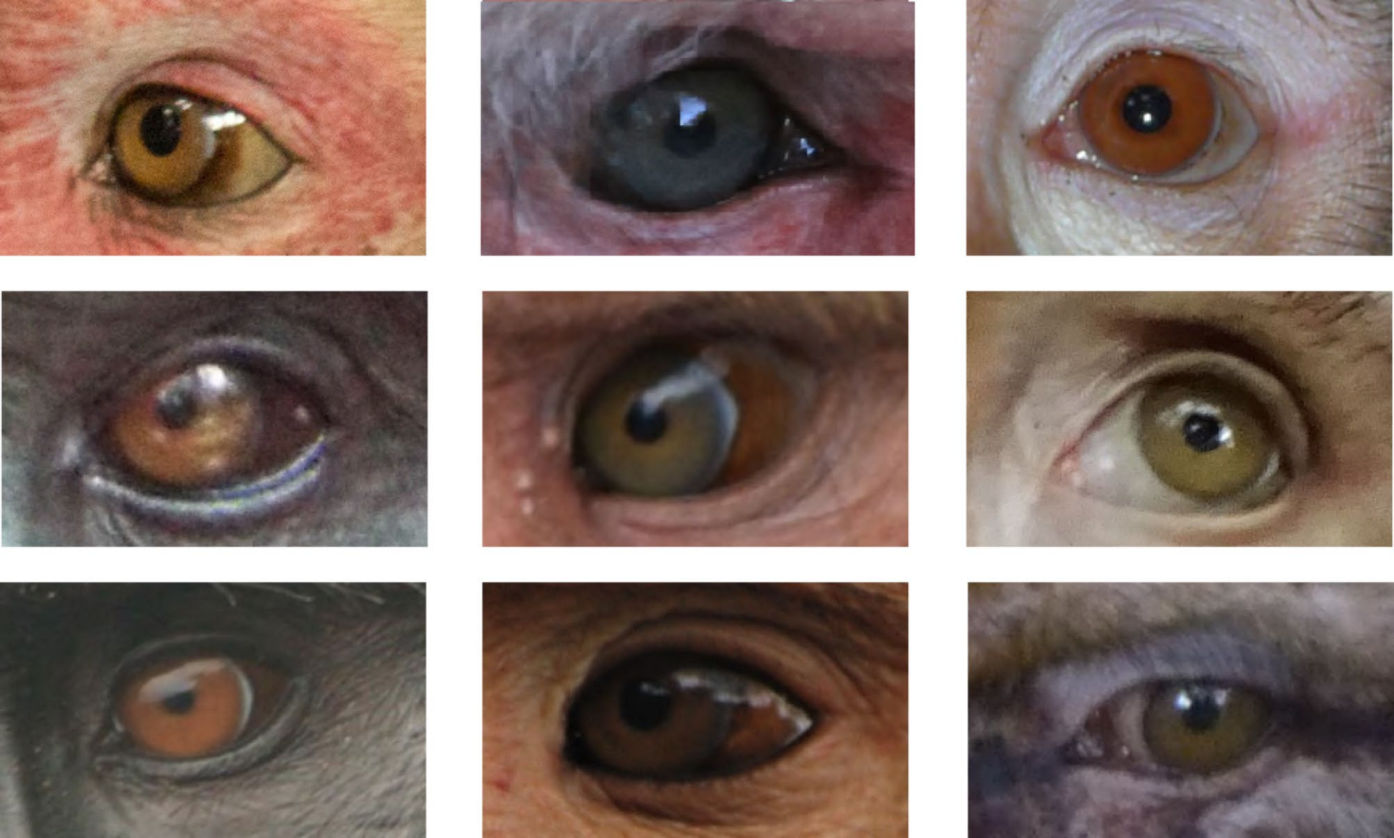
Macaque external eye appearance is extremely diverse. From left to right and top to bottom: *M. assamensis* (by Julia Ostner and Oliver Schülke), *M. fuscata* (by Alba Castellano-Navarro, Jorg J. M. Massen, Lena S. Pflüger and Pia M. Böhm), *M. leonina* (by Eva Gazagne, Aurélie Albert-Daviaud, and Juan Manuel José Domínguez) *M. maura* (by Víctor Beltrán-Francés), *M. mulatta* (by Stefano Kaburu), *M. nemestrina* (by Nadine Ruppert), *M. nigra* (by Jérôme Micheletta), *M. radiata* (by Shreejata Gupta), and *M. sylvanus* (by Bonaventura Majolo and Laëtitia Maréchal).

Presenting a wide variation in external eye appearance (Fig. 1), macaques are the most geographically widespread anthropoid primate genus (after humans;^16^) and the best described in terms of social structures, making this genus an ideal model to test hypotheses about the ecological and social drivers of eye color variation. Together with another species of papionin primate for which we had available on-site photographs (*Papio anubis*), we digitally quantified the eyes of nine macaque species. We used these measurements to test hypotheses about communicative and ecological functions driving eye coloration.

Macaques’ relatively well understood social structure allowed us to test the “gaze-camouflage” hypothesis. Some of the notions that the gaze-camouflage hypothesis are based on have been undermined by recent evidence (not all non-human primate eyeballs are cryptic; e.g. Kano et al.^17^ on chimpanzees). But, given that the gaze-camouflage hypothesis makes predictions about all primates, it is still valuable to examine its validity in other taxa. To test the gaze-camouflage hypothesis in macaques, we utilized the notion that different macaque species can be classified in terms of “social grade”^18^. A social grade of one denotes a species with steep dominance hierarchy, uni-directional patterns of aggression from more dominant to more submissive individuals, and little to no counter-aggression or reconciliation among non-kin. At the other extreme, grade four species are characterized by shallower dominance hierarchies, less skewed aggression, and greater reconciliation among non-kin following conflict. According to the “gaze camouflage” hypothesis, less tolerant (i.e., grade 1) species should have less conspicuous eye morphology to avoid triggering instances of physical aggression. According to the predictions of the “gaze camouflage” hypothesis, more tolerant (i.e., grade 4) species could have less cryptic morphologies, because the baseline risk of escalated conflicts is lower. It is also possible that male-male interactions drive variation in external eye appearance in macaques. Male macaques in troops containing males and females may engage in coalitionary aggression to compete for rank or access mates^19^. Thus, here we test whether there is a link between ocular conspicuity and different available constructs describing the social system of macaques.

Perea-García et al.^15^ proposed that depigmentation of peri-iridal tissues could be a by-product of selection against aggression. Because cutaneous melanocytes and the adrenal medulla derive from the same precursor cells in the neural-crest^20^, selection for tameness may result in pigmentation defects. The proposal by^15^, however, does not consider whether pigment-bearing tissues in the eye derive from the same precursors as those in the skin^21^. Recently^21^, cast doubt on the proposal by^15^ by comparing eyeballs of domesticates and their wild counterparts and finding no difference in brightness. Nonetheless, evidence is currently mixed^4^, calling for further investigation.

The widespread distribution of macaques makes them ideal to test the photo-regulatory functions of eye color diversity. Recent evidence suggests that primate eye morphology is related to environmental photopic factors^11^. For example, iris color shifts towards bluer hues farther away from the equator, suggesting that iris color helps primates regulate their circadian rhythm. The iris is not completely opaque and can allow some light to pass through depending on pigmentation levels. In highly pigmented eyes (brown), this contribution to intraocular light scattering is minimal, but in less pigmented eyes (lighter colors), the scattering effect becomes more pronounced^22^. Some of this light will stimulate intrinsically photosensitive retinal ganglion cells in the retina^23^. While several studies demonstrate that iris color affects visual function^24,25^, and that blue irises correlate with reduced incidence of seasonal affective disorder in humans^26^, a direct assessment of the influence of iridal coloration on the entrainment of circadian rhythm would be required to conclusively support said functions. Here, we test whether macaque irises become bluer farther from the equator. We also test whether latitude is related to levels of pigmentation of facial skin, Anterior Peri-Iridal Tissues (APIT), and Posterior Peri-Iridal Tissues (PPIT) between species, as pigmentation surrounding the iris was found to decrease with distance from the equator^11^. Previously, temporal-nasal asymmetries in the pigmentation of each eyeball in macaques were pointed out as suggestive of photoprotective functions^10^. Because of geometric properties of the face, more scattered light reaches the temporal quadrant of the eyeball compared to the nasal (Coroneo^27^), . However, because of the shape of the cornea, this scattered light is focused and beamed towards the nasal quadrant of the eyeball, which can receive up to 20 times the dosage of UV radiation compared to the temporal quadrant^27^. We quantitatively compared individual differences in pigmentation of the tissues adjacent to the iris (APIT) between the temporal and nasal regions of the eyeball to test whether exposure to sunlight increases pigmentation levels, as predicted by the photoprotective function^10,11^.

Lastly, studies inspecting primate eye morphology showed that there are within-species differences between sexes and age groups in levels of conjunctival (humans:^28^; orangutans:^14^; chimpanzees and bonobos:^15^; chimpanzees:^7^ and iridal pigmentation (chimpanzees and bonobos:^15^). Here, we compared our measurements of external eye appearance between age groups and sexes to explain the mechanisms underlying variation in pigmentation as well as developmental trajectories (Supplementary Materials).

## Materials and methods

### Samples

Photographs (*n* = 1121) were taken on-site by the authors of the study. Table S1 in the Supplementary Materials summarizes the devices and settings used to collect the samples. These photos were pre-selected from a much larger pool based on whether the different parts of the eye were visible. We selected photos that were of enough quality to distinguish all relevant parts of the eye with certainty, and in which the camera settings were appropriate for the lightning in which photographs were taken. This meant that the photo was of enough resolution to allow a clear visualization of the boundaries between different eye tissues; it was not blurry, and it was not obviously under- or overexposed. Our samples included the following species of the genus *Macaca* (*n* = 10): *M. assamensis*,* M. fuscata*,* M. leonina*,* M. maura*,* M. mulatta*,* M. nemestrina*,* M. nigra*,* M. radiata*,* M. silenus*,* M. sylvanus*. We also included photographs of *Papio anubis*, for which we only included photographs of adult individuals. A key aspect of our study relative to similar ones, that used photographs from the internet, is that we rely on samples of animals that actually inhabit the latitude values we use for our analyses. The inclusion of *P. anubis* allowed us to assess whether the patterns we observe in the macaque species in our sample are taxon-specific, or can be generalized beyond the genus *Macaca*. Two of our macaque species were represented by more than one population: *M. fuscata* (three populations) and *M. leonina* (three populations). Of the three populations of *M. fuscata*, one had been translocated from Japan (Minoo) to Austria (Affenberg Landskron). For this population, we report results when using the latitude of their population of origin in the main text. Results using the latitude of the location of translocation are included in the supplementary materials. For both *P. anubis* and *M. spp*, when multiple photographs were available for an individual, the average measurements of those photographs were used as an individual data point. Individual averages were obtained by summing up the values for each measurement for any given individual and dividing them by the number of photographs for that individual in the dataset (“*mean*” function in base R). This procedure resulted in an average of ~ 101 photographs per species. This number is well above the 12–14 samples per species recommended by^29^ to obtain reliable color measurements from uncalibrated pictures.

### Measurements

We sampled the eye on the left of each photograph, unless it was not visible or was hard to sample due to stark shadows, specular reflections, or other confounders. We used the PAT-GEOM plugin for ImageJ^30^ to measure brightness (in HSV) of the pupil iris, Anterior Peri-Iridal Tissues (APIT), and Posterior Peri-Iridal Tissues PPIT), and skin. The distinction between APIT and PPIT is relevant because the conjunctiva is typically the more deeply pigmented part of the tissues surrounding the iris, while pigment tends to fan out towards the posterior eyeball^1^. While in reality the entire anterior eyeball is covered by the conjunctiva, the portions of the conjunctiva distal to the iris are typically devoid of pigment, so the coloration is mostly due to the scleral tissues. Thus, our measurements aimed to approximate conjunctival and scleral pigmentation by differentiating between brightness measurements of APIT and PPIT, though there may not be an exact correspondence. Prior to measuring, images had to be separated in three channels, corresponding to Hue, Saturation, and Value (Brightness). To do the measurements, we used rectangles, the exact dimensions of which depended on each image. We extended a rectangle selection within the pupil for pupil measurements and noted the average brightness value for that selection. For iris measurements, this extended from the edge of the iris and pupil to the edge into the iris and surrounding tissues (avoiding e.g. limbal rings), or APIT. For measurements of the tissues proximal to the iris, the rectangle extended from the outer edge of the iris to the tissues distal from the iris, or PPIT, if visible. Otherwise, we selected the portion of the eyeball with the most visible peri-iridal tissues. Lastly, we used a rectangle to measure the tissues distal to the iris, which we identified as a noticeably depigmented portion of the eyeball most distal from the iris. The height of this rectangle was up to 10% of the iris diameter. The selection terminated whenever the rectangle reached a confounding factor (reflections or shadows), or if pigmentation changed abruptly (indicating a transition from the tissues proximal to the iris, to the tissues distal from the iris). For skin measurements, we sampled a rectangle immediately below the eye, of the same width and height as the eye, avoiding confounding factors (typically fur, specular reflections, or shadows). We did not measure PPIT if there was not a clear change in brightness along the tissues around the iris. Figure S8 illustrates how these cases affected whether tissues were sampled as APIT or PPIT. To compare nasal and temporal regions of eyeballs, we selected individuals with photographs in which nasal and temporal regions of the eyeball were visibly different and in similar lighting. We measured the temporal region starting from the edge of the iris into the adjacent tissues until pigmentation changed abruptly. We selected the same length of the nasal region of the eyeball (or until reaching confounding factors). We used measurements of brightness of the iris and adjacent tissues to calculate the difference in absolute measurements of brightness^11^ for each eye because of the issues^5,31^ pointed out regarding ratio-based measurements (like RIL). We also calculated the difference between measurements of brightness of the pupil and iris. We converted our hue measurements into RGB for ease of interpretation. Table S2 summarizes measurements for each population in the study. All data are available in the supplementary materials.

### Phylogenetic data

We used a consensus tree built with molecular data from the 10kTrees project (https://10ktrees.nunn-lab.org/*)* to account for phylogeny in our PGLS. The tree is accessible from the repository for this study.

### Statistical analyses

We used phylogenetic generalized least squares (PGLS). This method incorporates phylogenetic relatedness to account for non-independence of data points between the species in our sample^32^. As dependent variables in separate regression models (PGLS), we used measurements of brightness of the skin, iris, Anterior Peri-Iridal Tissues (APIT), and Posterior Peri-Iridal Tissues (PPIT), iris hue, and differences in brightness between pupil and iris, and iris and surrounding tissues. Latitudes of each population were noted down and used as a predictor in tests of photo-regulation. We used Latitude instead of UV index because the latter is a poor predictor of actual incidence of UV radiation in the eyes^33^. Species level conspecific lethal aggression data were obtained from^34,35^ respectively, and were used in tests of the self-domestication hypothesis. We noted down the social grade classification for each species from^18^. We used social grade 2 for *M. assamensis* instead of social grade 3, as OS considered this more accurate. Data on frequency of male-male coalitionary aggression was taken from^19^. These were used to test hypotheses about gaze camouflaging. For the latitude data, we made the values absolute to express distance from the equator. We examined diagnostic plots and applied Tukey Transformation when residuals were not normally distributed, or to improve homoscedasticity. Phylogenetic analyses vary in the number of species and individuals, depending on available data for each variable and species. Independent variables at the population level are summarized in the Supplementary Materials (Table S3). Analyses conducted in R (4.1.3). We used the gls function with Pagel’s *lambda* correlation structure^36^ from the nlme^37^ and ape^38^ package in R for our PGLS analyses. A full copy of the project, including the libraries, phylogenetic, tree, data files, and output from the *sessionInfo()* function can be accessed via the online repository (link in *Data accessibility statement*).

## Results

### Tests of the gaze-camouflage hypothesis

We ran two phylogenetic generalized least squares (PGLS) with social style grade at the species level as the predicting variable in the adult females in our sample (*n* = 587). In the first PGLS, we used the (Tukey-transformed) difference in measurements of brightness between iris and surrounding tissues^11^ as the outcome variable and found no relationship (intercept: 3.00, β = 0.057, SE = 0.284, t = 0.199, *p* = 0.842). In the second PGLS, we used the (Tukey-transformed) difference in measurements of brightness between the iris and pupil as the outcome variable and found no effect of social grade (Intercept: 6.138, β=-0.41, SE = 0.345, t=-1.187, *p* = 0.236; Figure S1). We ran two separate PGLS with the same outcome variables but frequency of male-male coalitionary aggression as the predicting variable, and using only the adult males in our sample (*n* = 517). Males of the different species covered the whole range of frequency of coalitionary aggression, including species like *M. leonina* and *M. nemestrina*, in which it was *never observed*, and species like *M. sylvanus* and *M. assamensis*, in which they are *routinely observed in all or most males* (coded 0 and 3 respectively in^19^. The first PGLS found no effect of frequency of male-male coalitionary aggression on the (Tukey-transformed) difference in measurements of brightness between iris and surrounding tissues (Intercept: 2.904, β=-0.014, SE = 0.249, t = 0.055, *p* = 0.956). The second PGLS also found no effect of male-male aggression on the difference in measurements of brightness between iris and pupil (Intercept: 23.929, β=-2.649, SE = 2.748, t=-0.964, *p* = 0.336; Figure S2).

### Tests of the self-domestication hypothesis

**Fig. 2:**
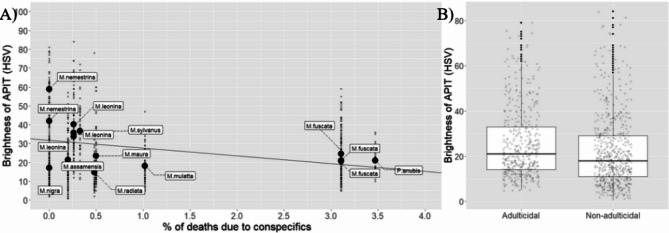
(**A**) Measurements of brightness (HSV) of the APIT regressed over rates of conspecific killing. Small dots represent individual measurements. Large dots represent population means. (**B**) Measurements of brightness of the APIT depending on whether adulticide has been recorded at the species level. Large dots represent outliers.

In the first test, we used the conspecific lethal aggression data used by^4^, originally from^34^. These data include a percentage of deaths due to conspecifics at the species level, and were available for all species in our sample, going from 0 to 3.47%. There was no effect of conspecific killing on (Tukey-transformed) brightness of the APIT (Intercept: 1.933, β=-0.048, SE = 0.065, t=-0.737, *p* = 0.462, Fig. 2A). Because these data include many instances of infanticide, they may not be the most appropriate to use as a proxy of temperament. When using the data from^35^, focused on adulticide, there was a small significant effect in the opposite direction to the expected, with adulticidal species displaying slightly brighter APIT (Intercept: 1.97, β=-0.231, SE = 0.107, t=-2.15, *p* = 0.032; Fig. 2B). We found, thus, no support for selection against aggression leading to lighter APIT.

### Tests of photo-regulatory functions

**Fig. 3:**
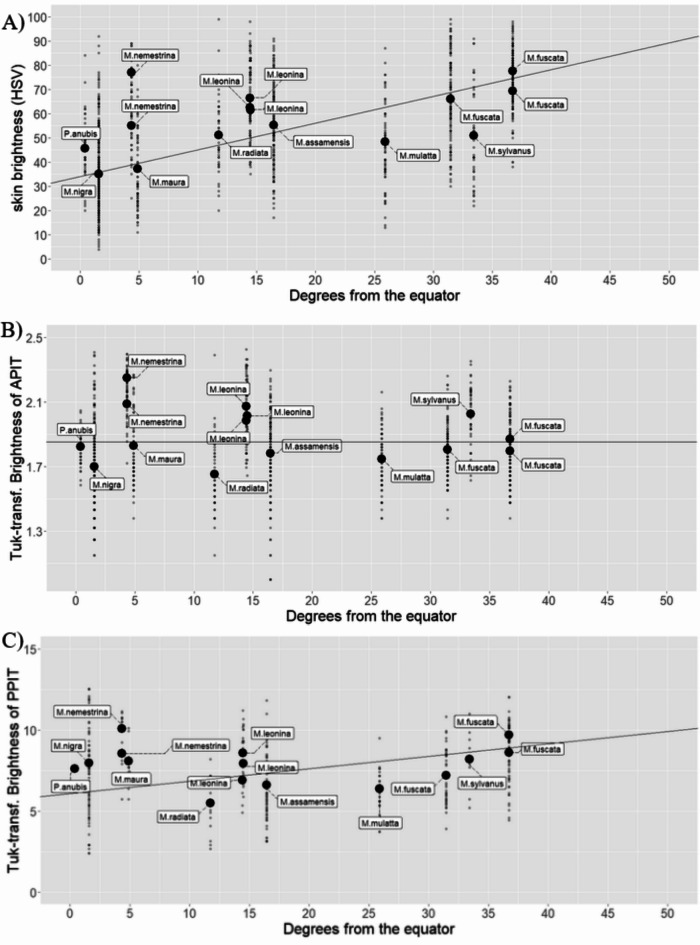
Measurements of brightness (HSV) of the (**A**) skin, (**B**) Anterior Peri-Iridal Tissues (APIT), and (**C**) Posterior Peri-Iridal Tissues (PPIT) regressed over distance from the equator. Small dots represent individual measurements. Large dots represent population means.

When using the values of the population of origin for the translocated *Macaca fuscata*, irises did not appear bluer with distance from the equator (Intercept: 0.234, β = 0.0001, SE = 0.001, t = 0.13, *p* = 0.896; Figure S3). When using the latitude values of the translocated location (Affenberg Landskron, Austria), the results were significant (Intercept: 0.22 β = 0.001, SE = 0.0006, t = 2.192, *p* = 0.029; Figure S4). There was a significant relationship between distance from the equator and measurements of skin brightness (Intercept: 34.177, β = 1.104, SE = 0.266, t = 4.156, *p* < 0.01; Fig. 3A), while there was no relationship between distance from the equator and measurements of (Tukey-transformed) conjunctival brightness (Intercept: 1.851, β = 0.00005, SE = 0.004, t = 0.014, *p* = 0.9891; Fig. 3B). Nonetheless, the result was significant when using the translocated values of latitude from Affenberg (Intercept: 1.779, β = 0.005, SE = 0.0023, t = 2.100344, *p* = 0.036; Figure S5). Finally, (Tukey-transformed) brightness of the PPIT significantly increased with distance from the equator (Intercept: 6.083, β = 0.076, SE = 0.038, t = 2.028, *p* = 0.0433; Fig. 3C). Results for brightness of the skin and PPIT remained in the predicted direction when including the translocated latitude from Affenberg, though their magnitude and significance changed slightly (full results in Table S4).

**Fig. 4:**
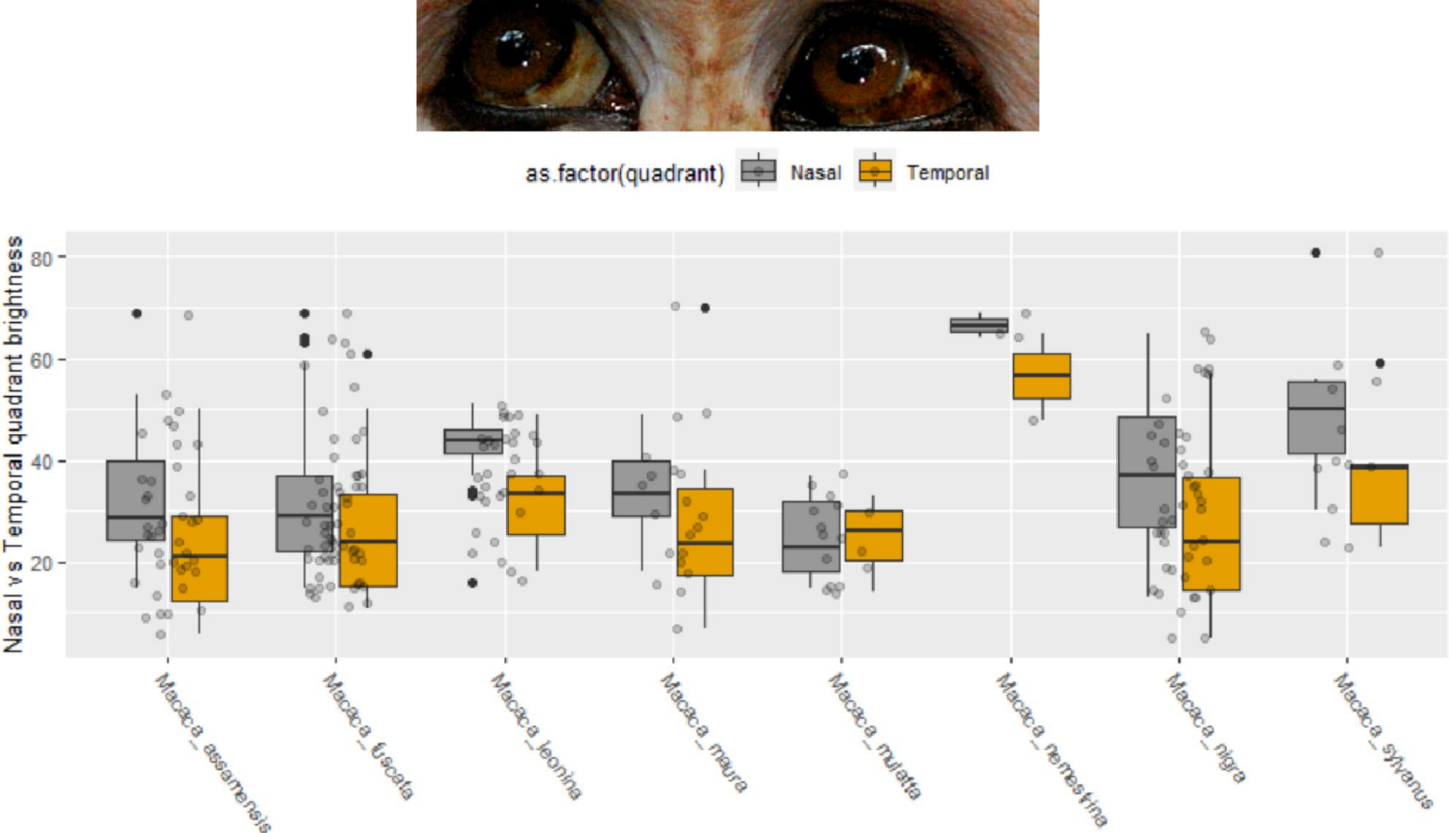
Measurements of brightness of the nasal (left eye) and temporal (right eye) quadrants of the conjunctiva in the eyes of the same individuals, in the same photograph. The figure shows raw measurements. The midline shows the median, and lower and upper hinges represent 25th and 75th percentiles, respectively. Black dots represent outliers. The eyeballs of a *M. assamensis* are on top, showing the differences between the nasal and temporal quadrants (photo by Julia Ostner and Oliver Schülke).

We measured the nasal and temporal quadrants of the APIT in photographs where both eyes of the same animal were clearly visible, the animal was facing the camera, and the animal was looking sideways (*n* = 115). This allowed us to formally test whether, as proposed in^10^, the temporal quadrant is consistently more pigmented (i.e., darker) than the nasal. The temporal quadrant was consistently darker than the nasal quadrant (Intercept: 5.196, β=-0.533, SE = 0.101, t=-5.268, *p* < 0.01; Fig. 4).

### Influence of exclusion of P. Anubis from analyses

Conceivably, the inclusion of *P. anubis* could obscure taxon-specific patterns pertinent to only macaques. To ensure that the patterns we report are indeed typical of macaques, and not due to the inclusion of a non-macaque species, we re-ran all the tests reported above excluding samples from *P. anubis*. These results are summarized in table S5. In short, results were qualitatively the same, and only very slightly quantitatively differed from those reported here.

## Discussion

We used digital measurements of different parts of the eye of a large number of adult individuals from fourteen populations of nine species of macaques, and another papionin species (*P. anubis*) to test hypotheses about communication and photo-regulatory functions as drivers of eye coloration. We also compared within-species differences between sexes, and between subadults and adults, to add to a growing number of descriptions of external eye appearance across primate species (Supplementary materials). There was no relationship between our measures of ocular conspicuity and social style grade or frequency of male-male coalitionary aggression, which we used as proxies of communicative functions. These results undermine the prediction of the “gaze camouflage” hypothesis that cryptic patterns of pigmentation are adaptive by reducing instances of physical aggression triggered by the perception of eye contact. Previous work showed no relationship between ocular conspicuity and canine size dimorphism^11^ - a reliable proxy of male intrasexual competition^39^, a context in which staring is expected to take place as a way to establish dominance^40^. Our results suggest that cryptic eye morphologies are not related to overall levels of intraspecific aggression, generalizing the results from^11^ to social interactions that extend beyond male intrasexual competition. Inspection of Figure S1 revealed that conspicuity in grades 1 and 4 is greater than in grades 2 and 3, suggesting an intriguing possibility: that the basal state of macaque external eye appearance is less conspicuous, but that species with very steep (grade 1) and very flat (grade 4) hierarchies benefit from conspicuous eyeballs. Grade 1 species could benefit from unambiguous staring, as a means to signal dominance (or submission, through averted gaze), avoiding costly aggression. Grade 4 species may employ gazing cues for affiliation or gaze following. Lastly, even though we tested the predictions of the “gaze camouflage” hypothesis, it should be noted that Kobayashi and Kohshima (2001) also considered that hiding gaze from predators could be adaptive. A study based on simulating the visual system of typical predators of a primate species (*Sapajus apella;*^41^ found evidence that suggests that this could be the case, though it should be noted that this study is not based on experimental data and makes crucial assumptions about whether birds of prey attend to the eyes of the animals they hunt. Thus, while we consider that the literature has accumulated evidence undermining the “camouflage hypothesis” with regards to conspecifics, this is not so with regards to predators. It is important to note, however, that our method assumes that macaque visual perception is functionally similar to that of humans, and that there is little variation across macaque species. Methods like those deployed by^41,42^ that simulate visual systems, are more adequate for questions concerning perception. In short, we found no support for the idea that eyeball detectability decreases in more despotic intolerant social structures, but if a conspicuous gaze serves in both highly competitive and cooperative contexts, a non-linear relationship may more adequately describe a putative link between eyeball detectability and social structures, and it could be that camouflaging gaze is adaptive towards predators, rather than conspecifics.

We further explored the hypothesized relationship between selection against aggression and depigmentation of APIT^4,15,20^ by using percentage of deaths by conspecifics as a proxy of selection for aggression, but could not replicate^4^ results. With another dataset focused on adulticide^35^, our results went against the predictions, with adulticidal species displaying lighter APIT. Our results support^21^’s conclusions that peri-iridal depigmentation is not a correlate of selection against aggression, but are at odds with^4^ conclusions, who found that peri-iridal pigmentation was darker in species with greater percentage of deaths due to conspecifics. Given that Caspar et al.^21^ sampled actually domesticated species and their wild counterparts, we are inclined to agree with their^21^ conclusion. Assuming that pigmentation in APIT^1^ has an adaptive value, it would be unreasonable to expect detectable differences in the depigmentation of tissues surrounding the iris across a diverse, ancient lineage of natural species like primates, if these differences were not adaptive. Over evolutionary time, the selective pressures favoring individuals with pigmentation would likely outweigh the initial developmental constraint linking reduced pigmentation—caused by selection against aggression—with a non-adaptive outcome^20^. Important methodological issues in^4^ study (small sample size; imprecise measurements that may have included e.g. temporal wedge in the sampling area for conjunctival pigmentation measurements) reinforce our inclination to conclude that depigmentation in the tissues around the iris is not a correlate of selection against aggression (contra e.g. Perea-García et al.^15^, and therefore is unlikely to act as an honest indicator of a wild animal’s temperament^43^.

Irises did not appear bluer farther from the equator when using the latitude values corresponding to the population of origin of the *M. fuscata* translocated to Affenberg (Austria). These results are not in line with^11^, who found that the irises of 76 species of anthropoid primates shifted to green-blue with distance from the equator. The species in our sample represent, to the naked eye, the typical gamut of primate iris colorations - from amber to green-blue. Furthermore, the distribution of the species in our samples comprehends most of the latitudinal variation present in extant primates. It is thus puzzling that the pattern observed by^11^ is not replicated here. Zhang & Watanabe^44^ were the first to provide preliminary evidence that the frequency of blue eyes in Japanese macaques increases in more northern populations. It could be that this pattern is more pronounced within *M. fuscata* than it is at the genus level. Nonetheless, the choice to assign the latitude values of the original population from which the Affenberg population descends builds on the assumption that external eye appearance is rather fixed at the species level, and that it is unlikely to change in the course of a few generations. The ambiguous results for the circadian rhythm hypothesis^10,11^ in our results calls for further investigations, opening the possibility that processes such as sexual selection play a role in determining iris color as has been proposed in humans^45^. Another possibility is that individuals in the translocated population, who inhabit an environment with less UV radiation, do not develop as much pigment throughout their lifetimes. While one may assume that external eye appearance is fixed at the species level and tightly constrained by genetics, at least one twin study shows that those living farther from the equator display lighter irises than their counterparts living closer to the equator^46^. Thus, it is plausible that, similar to skin in humans, there is some degree of phenotypic plasticity in iridal pigmentation. However, other possibilities could explain the patterns we observed - for example, it could be that a bottleneck or drift in the population in Affenberg gave rise to the results. These results, while pointing in interesting directions, should thus be taken with caution.

The relationship between measurements of skin brightness and latitude suggests that dermal pigmentation in macaques is subject to similar selective pressures as in humans^12^. Human skin pigmentation has been explained as responding to two clines: first, to protect the skin from UV radiation, as populations near the equator have been selected for increased pigmentation; second, populations with more northern distributions have been selected for decreased pigmentation to enable vitamin D_3_ synthesis in low UV-B environments^12^.

We found a positive relationship between brightness measurements of PPIT and latitude. The strong effect of latitude on pigmentation of the skin and PPIT lends support to the idea that photoprotection drives patterns of pigment distribution in the eyeballs of primates. The lack of relationship between pigmentation in the APIT and latitude is hard to explain. It may be that, in macaques, the pressure to protect the eyeball from sunlight is reduced, so that variation in pigmentation is due to other functions (e.g. communication, sexual selection) or that it is under relaxed selection. A relaxation in the need to protect the eyeball from sunlight may come about due to multiple factors - for example, in humans, the lack of lateral epithelial stem cells in the corneal limbus could allow for reduced pigmentation of the eyeball^10^. It would be especially interesting to examine *Macaca nemestrina*, who display the greatest levels of depigmentation in all measurements in our dataset, despite being one of the species living the closest to the equator. *Macaca fuscata* is also interesting in this respect, showing considerable levels of pigmentation in the APIT despite its distribution being farthest from the equator. The case of *M. fuscata* may be due to their inhabiting land with considerable snow cover for months^47^. Ground cover reflectivity is the most important factor determining irradiation to the eyeballs, and the most intense reflection from ground cover described is fresh snow (88%;^48^. Perhaps taking into consideration the reflectivity of the typical ground cover in the habitat of *M. nemestrina* could help explain their unusual eye appearance for an equatorial species. Behavioral differences such as a preference for high vegetation cover or low canopy may also reduce exposure to sunlight, leading to reduced selection pressures. Indeed, while there is a trend towards greater pigmentation closer to the equator in the only previous study looking at the influence of photopic factors in primate ocular coloration, many species do not seem to follow that trend^11^. Thus, while ambient light appears to be an important factor contributing to variation in patterns of ocular coloration, ground reflectivity is likely an important factor that has yet to be accounted for. Once again, however, assigning latitude values from the original population to the translocated population in Affenberg makes only sense under the assumption that ocular pigmentation shows no phenotypic plasticity. With the translocated values, results were significant and in the predicted direction. While these results could suggest phenotypic plasticity in ocular dermal melanocytes, they should only be taken as preliminary until further tests. Lastly, there are differences in the environment of the Affenberg population relative to their population of origin in Minoo (Japan), beyond their geographical location. The animals in Affenberg are part of an entertainment enterprise where they are provisioned, they are regularly exposed to human visitors, and they do not have opportunities to interact with other groups. However, it is hard to compare to what extent this is different from their population of origin - the population in Minoo is also partly provisioned, and there is the possibility that the population in Affenberg experiences reduced predation pressure, though no data are available to conduct quantitative comparisons^49^. Preliminary evidence suggests that situations of commensalism where food is provided and predation risk eliminated could lead to the development of traits associated with domestication in mice, crucially including depigmentation of their coat, in a few generations^50^. An alternative interpretation of the brighter APIT and PPIT measurements in the Affenberg population could be that it is the relaxation of stressors (foraging, competition for resources and mating opportunities, and risk of predation) that has resulted in a domestic-like phenotype. However, the results and discussion in^21^ weaken the proposal that selection against aggression could result in depigmentation of tissues in the eyeball. Our own tests of the self-domestication hypothesis further undermine the only previous result that supported a link between ocular depigmentation and selection against aggression. Thus, we are more partial to interpreting the slightly lighter APIT of the population in Affenberg compared to their population of origin in Minoo (see table S5) as deriving from the different photopic conditions of their environment.

The similarity in results including and excluding the data from the *P. anubis* population (Table S5) suggests that the results we found are not specific to macaques, and that the within-genus patterns we found are robust. The measurements of *P. anubis* in our sample, as well as their latitudinal values, are very similar to those of *M. nigra* and *M. maura* (Tables S2 and S3). At the same time, the phylogenetic divergence of *P. anubis* is relatively small with regards to *Macaca spp*. Therefore, it would be surprising if its inclusion substantially altered our results. Given that similar patterns were found in a study examining a broader taxonomic sample including most genera of anthropoid primates^11^, it is also expected that the inclusion of a species outside the genus *Macaca* would not alter our results in a substantial manner.

We found strong within-individual differences in pigmentation between nasal and temporal regions of the eyeball, suggesting that the distribution of pigment in the eyeball responds to the so-called “Coroneo effect” of limbal focussing^48^ as proposed by^10^. Absorbing as much light as possible on the temporal quadrant will prevent ophthalmhelioses on the nasal quadrant. In humans, the nose bridge reflects substantial UV radiation into the nasal side of the eyeball^51^, but this is unlikely to be as important in other primates, whose snouts are not protruding at the height of eyeballs. It would be interesting to examine patterns of peri-iridal pigmentation in humans, for whom reflection from the nose may be an additional source of phototoxicity. Because the mechanisms of exposure geometry and spectral environment on the human eye are relatively well understood^52^, there is promise in integrating knowledge from ophthalmology and eye care into evolutionary investigations of the drivers of variation in external eye appearance. In short, intra-individual variation in peri-iridal pigments appears driven by photoprotective pressures.

Our method depends on evening out random variability due to ambient lighting by using a large number of photographs. This approach has been validated in^29^. We believe methods such as those employed in^41,42^ have the potential to obtain more accurate values because^41,42^ standardize their samples by using a color swatch^53^, thus minimizing error in measurements due to ambient lighting. Additionally, their method simulates the visual system of relevant species to the ecology of their subjects (be it conspecifics or e.g. predators), while ours assumes enough similarity between that of humans and macaques. Nonetheless, our method shows comparability across studies using the same method as well as comparability with studies using methods like those employed by^41,42^. To address concerns regarding the validity of data obtained with the method we use here in more detail, we have included a dedicated section at the end of the Supplementary Materials.

To conclude, our study did not find that measures of ocular conspicuity (conjunctivo-iridal and pupillo-iridal contrast) were related to proxies of steepness of dominance hierarchy (social style grade) nor intraspecific aggression (male-male coalitionary aggression), failing to support the gaze camouflage hypothesis. We found no evidence in favor of the self-domestication hypothesis when comparing levels of depigmentation of the APIT across species with different measures of conspecific lethal aggression. Our analyses could suggest that iris color shifts towards blue farther from the equator, and that APIT becomes more lightly pigmented with distance from the equator. While these results would be in line with the observed in other primate lineages^11^, they should be taken as preliminary, due to the difficulty of interpreting results from translocated populations. If these results were an artifact, this would suggest that the iris and APIT are under pressure to comply with functions other than photo-regulation, or under relaxed selection. Alternatively, it may be that latitude alone is insufficient and factors such as ground and vegetation cover are important. In contrast, both PPIT and skin become more pigmented in animals closer to the equator, suggesting photoprotective functions of variation in pigmentation of the eyeball. This concords with our finding that the temporal side of the eyeball, which is typically more exposed to scattered sunlight, is more pigmented than the nasal side of the eyeball in the same individuals. These results add to the investigations supporting a role of photoprotective, but not communicative functions in driving patterns of eye coloration among anthropoid primates.

## Electronic supplementary material

Below is the link to the electronic supplementary material.

Supplementary Material 1

## Data Availability

Measurements, species-level data, and R code available here: https://osf.io/ytxnw/?view_only=dae93ae637fc441b88d410fe170172e2.
