# Supplementary material for: Photoregulatory functions drive variation in eye coloration across macaque species
Source: Sci Rep. 2024 Nov 24;14:29115. doi: 10.1038/s41598-024-80643-4 (PMC11586437; doi:10.1038/s41598-024-80643-4)

SUPPLEMENTARY MATERIALS

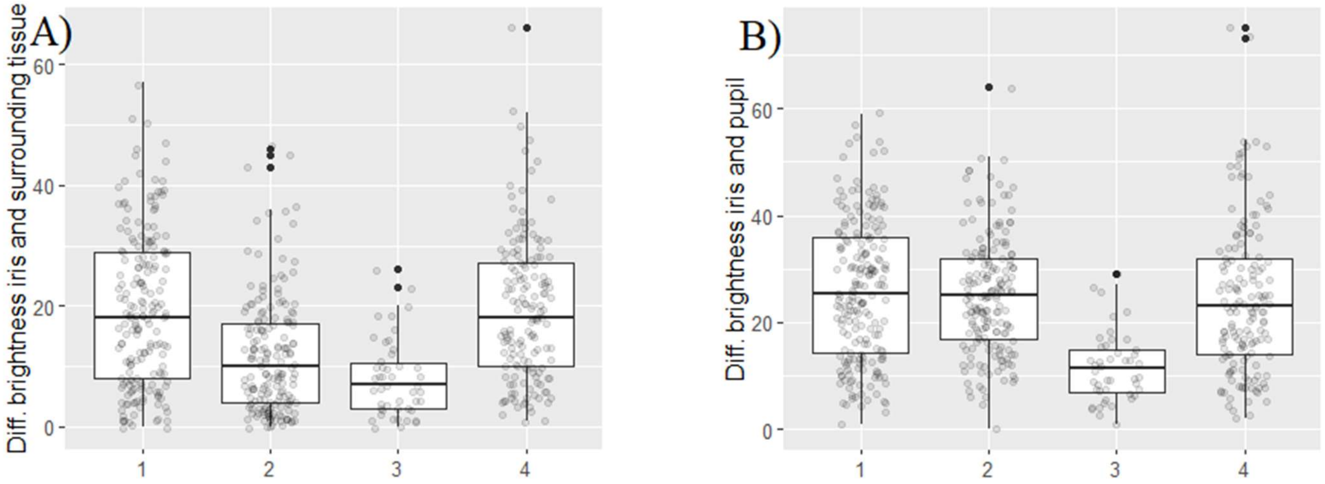

Figure S1.- No relationship between social style grade (X-axis) and A) difference in measures of brightness of the iris and adjacent tissue, or B) difference in measures of brightness of the pupil and iris. These analyses include only measurements from females in our sample. The figure shows raw measurements. The midline shows the median, and lower and upper hinges represent 25th and 75th percentiles, respectively. Black dots represent outliers.

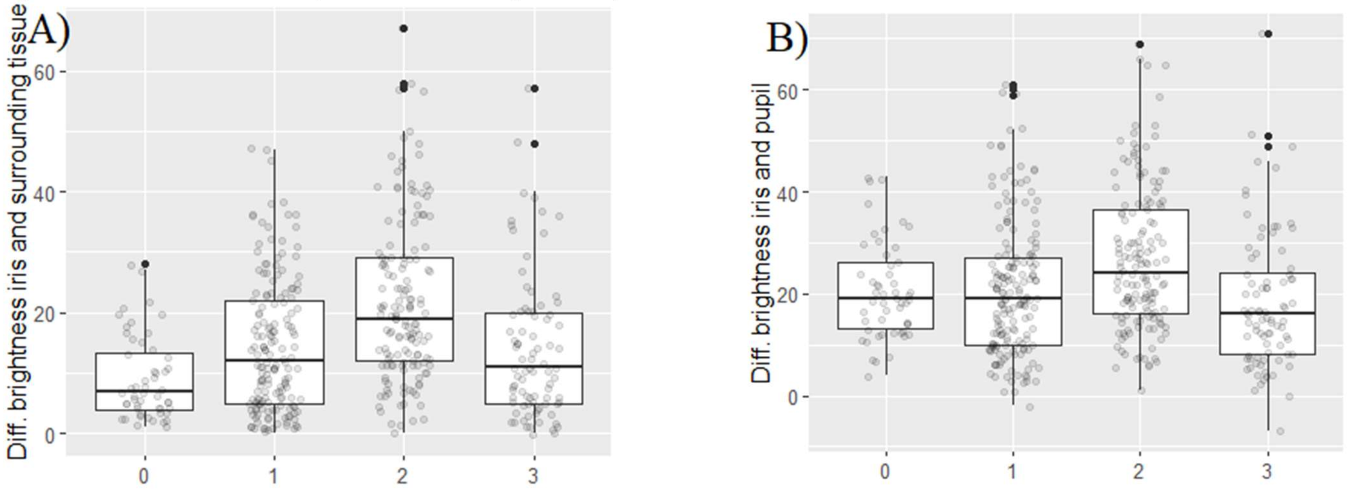

Figure S2.- No relationship between frequency of male-male coalitionary aggression (X-axis) and A) difference in measures of brightness of the iris and adjacent tissue or B) N difference in measures of brightness of the pupil and iris. These analyses include only measurements from males in our sample. The figure shows raw measurements. The midline shows the median, and lower and upper hinges represent 25th and 75th percentiles, respectively. Black dots represent outliers.

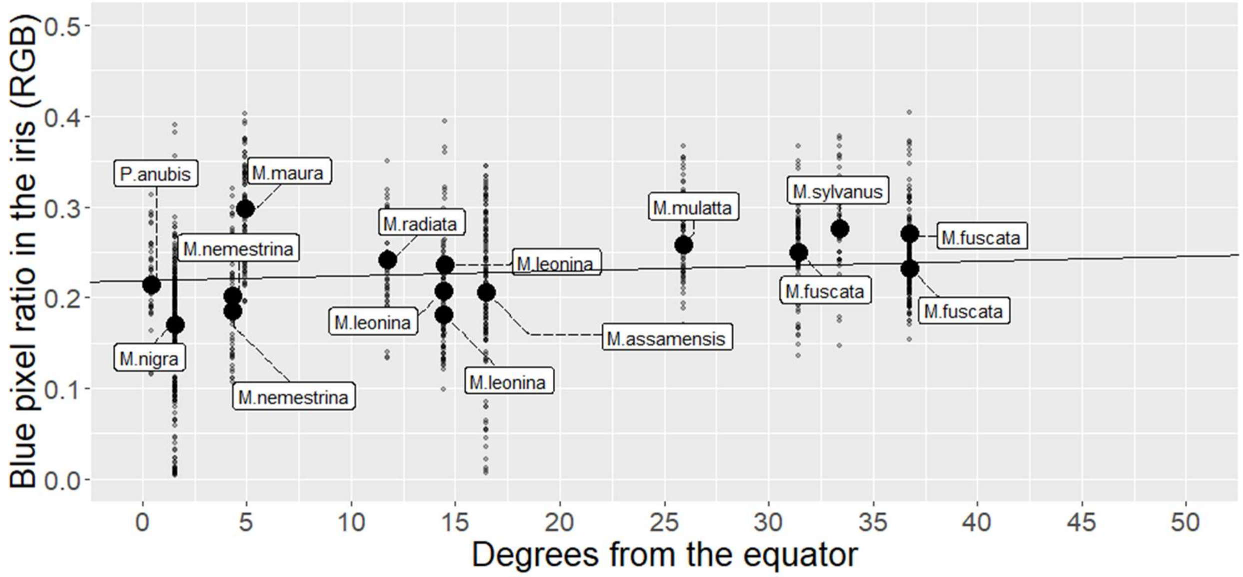

Figure S3.- Irises do not reflect more blue light in species distributed further from the equator. This figure takes the latitude values of the population of origin for the Affenberg population. Small dots represent individual measurements. Large dots represent population means.

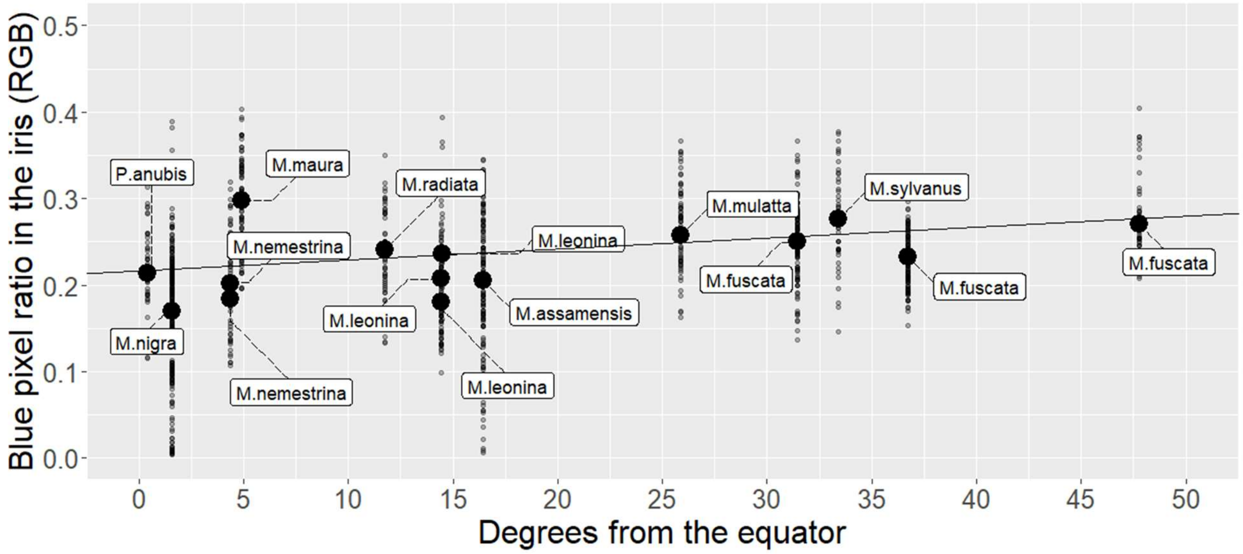

Figure S4.- Figure showing the regression of measurements of proportion of blue pixels in the iris over latitude when including the translocated latitude from Affenberg

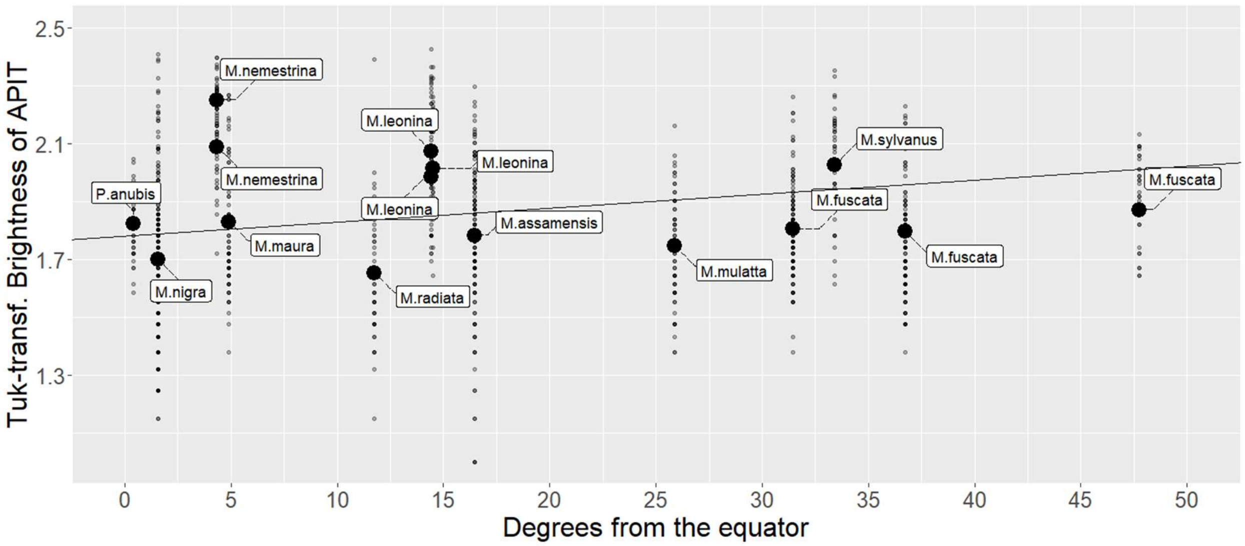

Figure S5.- Figure showing the regression of (Tuk. transformed) brightness measurements of the anterior peri-iridal tissues over latitude when including the translocated latitude from Affenberg

Table S1.- Cameras and settings used to take photographs

**Population**	**Camera(s)**	**ISO**	**F**	**Exposure time**	**Focal length**	**Flash**
Papio anubis	Canon EOS 400D	100	variable	variable	200mm	No
Macaca maura	Fujifilm FinePix S8500	variable	f/6.5	variable	198mm	No
Macaca nemestrina	NIKON COOLPIX S6400, SONY DSC-HX60V, OLYMPUS SP800UZ	variable	variable	variable	variable	No
Macaca radiata	NIKON D40	variable	variable	variable	200mm	No
Macaca assamensis	NIKON D5000, NIKON D7000 NIKON D70s, Canon EOS Kiss Digital X, Canon DIGITAL IXUS 95 IS, FUJIFILM FinePix S6500fd	variable	variable	variable	variable	variable
Macaca leonina	Panasonic DMC-FZ200, Canon PowerShot SX1 IS, DMC-FZ18	variable	variable	variable	variable	variable

Macaca nigra	Canon EOS 400D DIGITAL, Canon EOS 350D DIGITAL, Canon EOS 50D, Canon EOS 500D	variable	variable	variable	variable	No
Macaca sylvanus	FUJIFILM FinePix S5700 S700, FUJIFILM FinePix T200, NIKON D40X	variable	variable	variable	variable	No
Macaca fuscata (Affenberg)	Canon EOS 6D	10000	variable	variable	400mm	No
Macaca fuscata (Koshima)	NIKON D3200	variable	variable	variable	variable	No
Macaca fuscata (Minoo)	NIKON COOLPIX B700	variable	variable	variable	variable	No
Macaca mulatta	NIKON D3300, Canon EOS REBEL T1i	variable	variable	variable	variable	No

Table S2.- Summary of measurements by population

**Population**	**PupilB**	**IrH**	**IrS**	**IrB**	**ConjunctivaBrightness**	**Sclera Brightness**	**skin**	**HC**	**pupHC**	**red**	**green**	**blue**	**BlueProportion**
M. assamensis	10.11	43.01	50.07	33.11	21.37	31.14	55.17	14.82	23.00	83.84	67.83	41.84	0.21
M. sylvanus	18.84	89.41	33.07	29.89	36.61	45.78	50.93	8.09	11.05	74.09	60.70	50.86	0.28
M. radiata	9.44	33.62	42.70	23.48	14.86	22.07	51.07	9.07	12.80	59.69	43.67	32.63	0.24
M. fuscata (Minoo)	10.39	30.19	45.65	43.30	20.98	50.05	77.71	22.69	32.91	110.39	82.88	58.33	0.23
M. fuscata (Affenberg)	9.87	65.06	32.34	31.71	24.50	62.10	69.45	9.09	22.14	78.31	72.84	56.51	0.27
M. fuscata (Kojima)	16.80	44.84	39.70	42.37	20.60	36.32	66.03	21.90	25.57	106.86	87.08	64.63	0.25
M. leonina C	15.18	23.55	62.88	41.32	40.12	49.69	66.39	7.02	26.14	105.33	66.96	42.11	0.18
M. leonina B	13.41	60.93	45.99	31.51	35.68	43.15	61.62	7.08	18.10	79.08	59.86	44.21	0.24
M. leonina A	12.54	33.99	52.53	35.15	33.78	33.57	62.54	8.99	22.62	89.58	69.66	42.00	0.21
M. maura	20.11	126.77	27.48	32.93	23.53	44.64	37.32	13.6	12.82	80.51	71.66	62.67	0.30

								7					
M. nemestrina B	13.80	27.65	54.92	33.05	42.02	49.55	55.05	11.06	19.28	84.03	59.85	39.96	0.20
M. nemestrina A	23.28	31.47	60.55	53.23	58.89	66.62	76.97	8.17	29.95	135.26	94.24	53.49	0.18
M. mulatta	9.59	48.06	36.63	23.41	18.09	29.12	48.47	7.12	13.78	59.43	47.94	37.47	0.26
M. nigra	9.94	34.45	63.44	37.25	17.16	43.46	35.14	21.40	27.25	94.80	67.16	34.66	0.17
P.anubis	16.80	24.02	52.88	46.88	20.97	40.00	45.62	27.12	29.68	119.40	79.23	52.73	0.21

Table S3.- Population-level data

Species	Location Lat, Long	Location original population (used in the study)	Grade (females)	frequency male-male coalition	Conspecific Killing (Gómez et al., 2016)	Conspecific Killing (Gómez et al., 2021)
M. assamensis (Oliver & Julia)	16.450000, 101.633333	16.450000, 101.633333	2	3	0.2	0
M. fuscata (Alba Castellano Navarro)	31.453195, 131.376314	31.453195, 131.376314	1	1	3.11	1
M. fuscata (Jorg J. M Massen)	47.762395, 9.245170	36.73016227450577, 136.0098352623713 5	1	1	3.11	1
M. fuscata (Lena Pflüger & Pia Böhm)	36.73016227450577, 136.00983526237135	36.73016227450577, 136.0098352623713 5	1	1	3.11	1
M. leonina (Aurélie Albert)	14.439742, 101.371834	14.439742, 101.371834	2	0	0.26	0
M. leonina (Eva Gazagne)	14.487393572447719, 101.9093731349615	14.48739357244771 9, 101.9093731349615	2	0	0.26	0
M. leonina (Juan Manuel José Domínguez)	14.430383493275654, 101.37366361070754	14.43038349327565 4, 101.3736636107075 4	2	0	0.26	0
M. maura (Victor Beltrán)	-4.906073, 119.778637	-4.906073, 119.778637	4	NA	0.5	0
M. mulatta (Stefano Kaburu)	25.89125301004336, 91.10413368003817	25.89125301004336, 91.10413368003817	1	1	1.02	1
M. nemestrina (Nadine Rupert)	4.346924091416186, 100.61998865739352	4.346924091416186, 100.6199886573935 2	2	0	0	1

M. nigra (J. Micheletta)	1.568370, 125.162562	1.568370, 125.162562	4	2	0	0
M. radiata (Shreejata Gupta)	11.755686799313958, 76.44380697630986	11.755686799313958, 76.44380697630986	3	1	0.48	0
M. sylvanus (Bonaventura Majolo & Laetitia Marechal)	33.419941061699, -5.178380121084515	33.419941061699, -5.178380121084515	3	3	0.33	1
P. anubis (Marie Bourjade)	(latitude 0.4040020; 0 24014.41 N; longitude 37.1644840; 37 9052,14 E)	(latitude 0.4040020; 0 24014.41 N; longitude 37.1644840; 37 9052,14 E)	NA		3.47	1

Table S4.- Full results of the regression of measurements of brightness of the skin and PPIT over latitude when including the translocated latitude from Affenberg

	Intercept	β	Std. Error	t	p
skin	44.58622	0.39900	0.151904	2.626698	0.0087
PPIT (Tuk. transformed)	5.58996	0.106354	0.0268346	3.963328	0.0001

Sex and Age Group differences

To inspect differences between the sexes in our sample, we averaged measurements by individual (n=576) of all adult individuals in our sample (n=1121) and used these averages to run separate two-way ANOVAs for each of our measurements, with sex, species, and their interaction as predictors. There was a significant difference between measurements of brightness of the APIT by sex (F=7.129, p=0.008), with females having slightly lighter APIT in most species. The difference was not significant for species (F=3.5, p=0.062), nor the interaction between sex and species (F=0.111, p=0.739). For measurements of brightness of the PPIT, there was a difference between species (F=6.657, p<0.01) but no significant difference between sexes (F=1.339, p=0.248) nor their interaction (F=1.777, p=0.082). We also found females tended to display lighter irises (F=14.722, p<0.01), and that the brightness of the iris was different between species (F=17.564, p<0.01), but the interaction between sex and species was not significant (F=1.418, p=0.177). Females again displayed lighter measurements of skin brightness (F=12.479, p<0.01) and we found differences between species (F=53.082, p<0.01), but there was no significant interaction between sex and species in skin brightness (F=0.337, p=0.963; Figure S6).

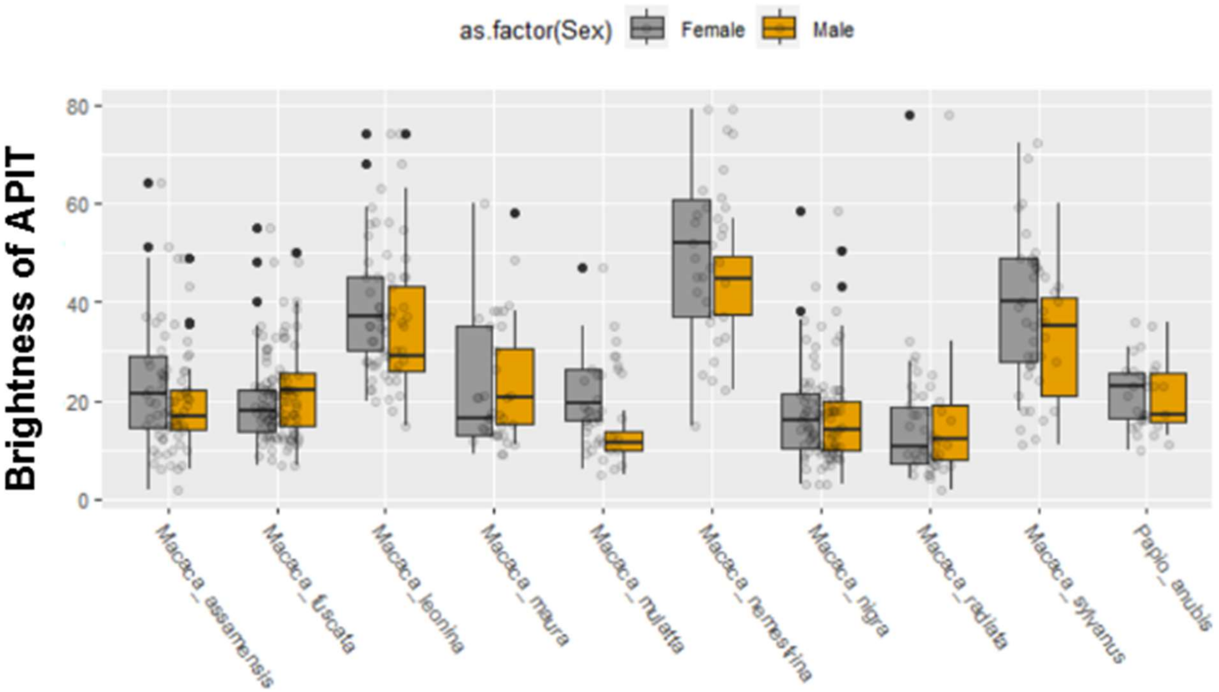
as.factor(Sex)
Female
Male
Brightness of APIT
0
20
40
60
80
Macaca_assamensis
Macaca_fuscata
Macaca_leonina
Macaca_maura
Macaca_mulatta
Macaca_nemestrina
Macaca_nigra
Macaca_radiata
Macaca_sylvanus
Papio_anubis

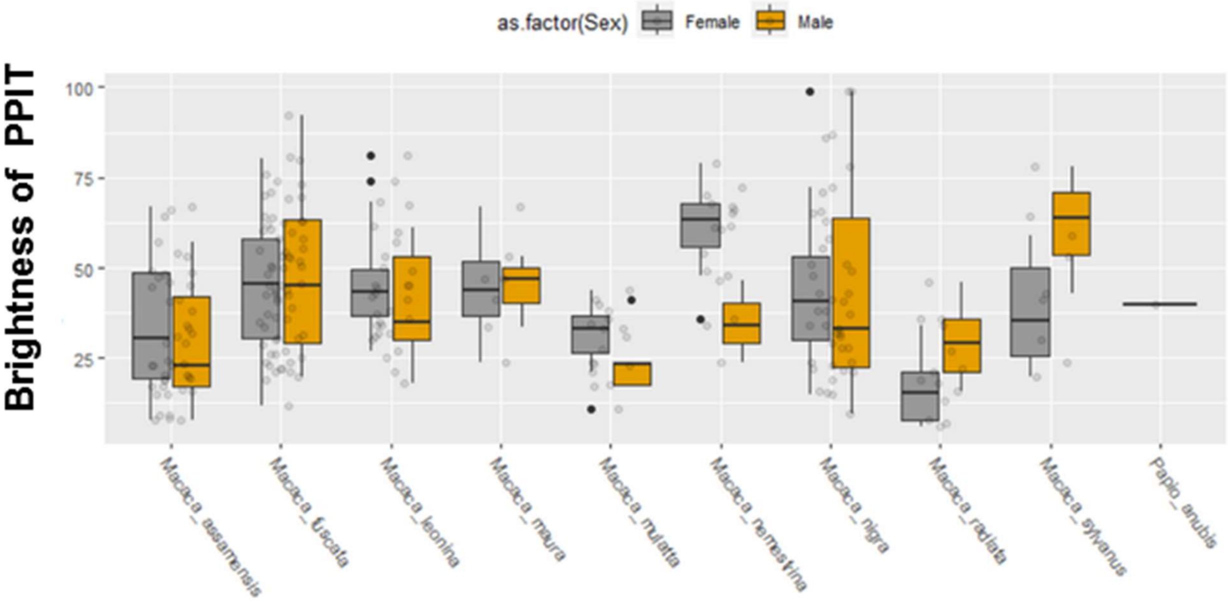
as.factor(Sex)
Female
Male
Brightness of PPIT
25
50
75
100
Macaca_assamensis
Macaca_fuscata
Macaca_leonina
Macaca_maura
Macaca_mulatta
Macaca_nemestrina
Macaca_nigra
Macaca_radiata
Macaca_sylvanus
Papio_anubis

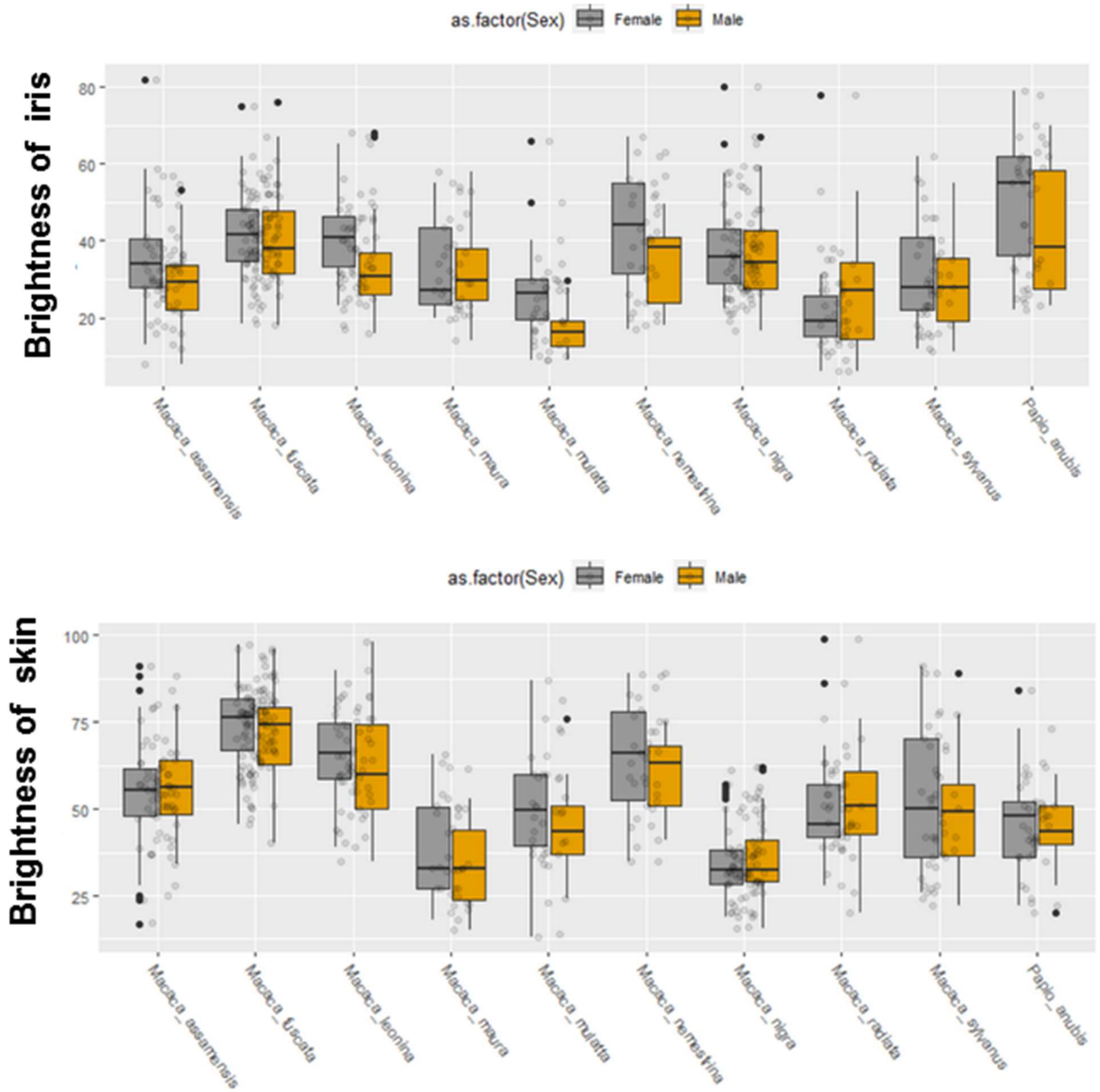

Figure S6.- Measurements of brightness of the APIT, PPIT, iris, and skin by species and sex. The figure shows raw measurements. The midline shows the median, and lower and upper hinges represent 25th and 75th percentiles, respectively. Black dots represent outliers.

Lastly, to understand whether developmental progression altered the brightness of the different parts of the eye, we inspected differences in our measurements between infants and sub-adults (7 years and younger) and adults (older than 7 years) in all species in our sample that included photographs of subadults (n=6). To do this, we averaged measurements by individual (n=729) of all individuals in our sample (n=1484) and used these averages to run separate two-way ANOVAs, with age category, species, and their interaction as predictors. Sub-adults had considerably lighter APIT (F=77.593, $p<0.01$), and we found significant differences between

species (F=5.079, p=0.025), but the interaction between age group and species was not significant (F=0.651, p=0.412). Sub-adults also had lighter sclerae (F=8.201, p<0.01), and we found significant differences between species (F=7.223, p<0.01) and in the species-age group interaction (F=3.669, p<0.01). There were no significant differences in the brightness of the iris between age categories (F=1.622, p=0.203), but there were differences between species (F=20.974, p<0.01) and their interaction (F=2.861, p=0.015). Sub-adults also had lighter skin (F=31.126, p<0.01), and there were significant differences between species (F=60.517, p<0.01), and the species-age category interaction (F=6.176, p<0.01; Figure S7).

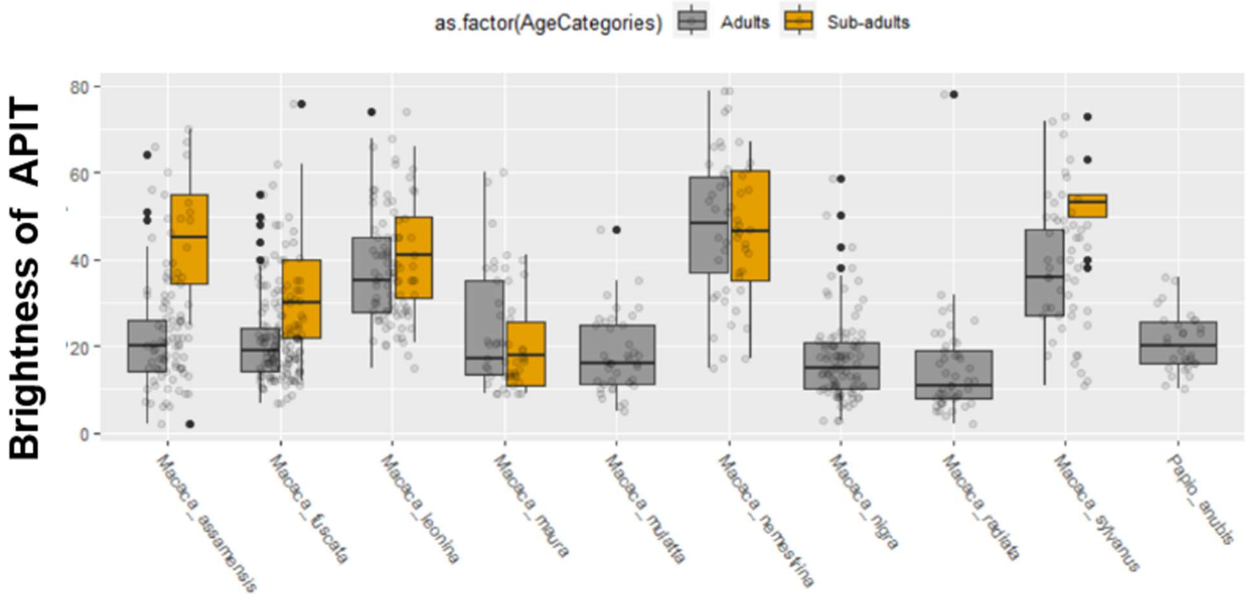

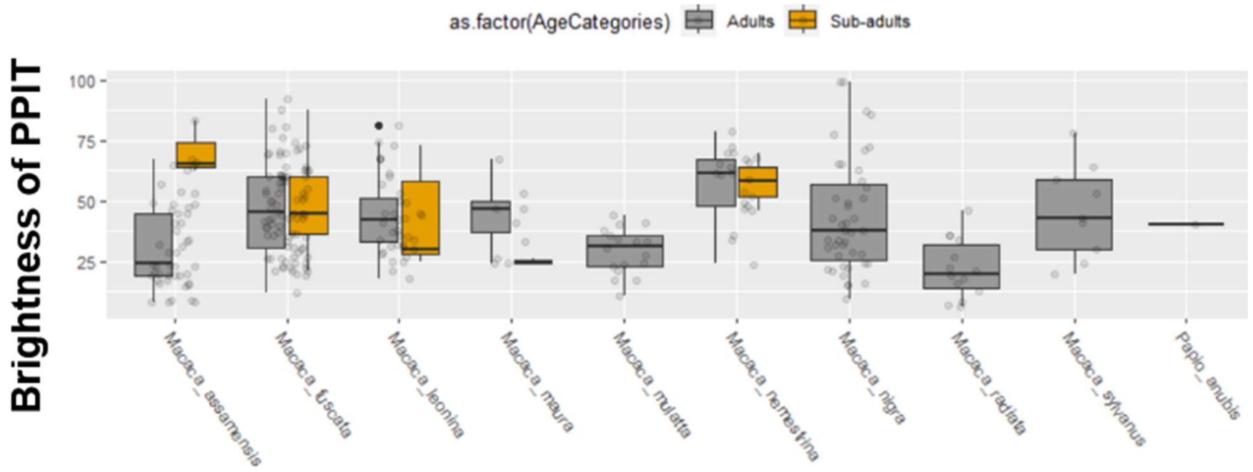

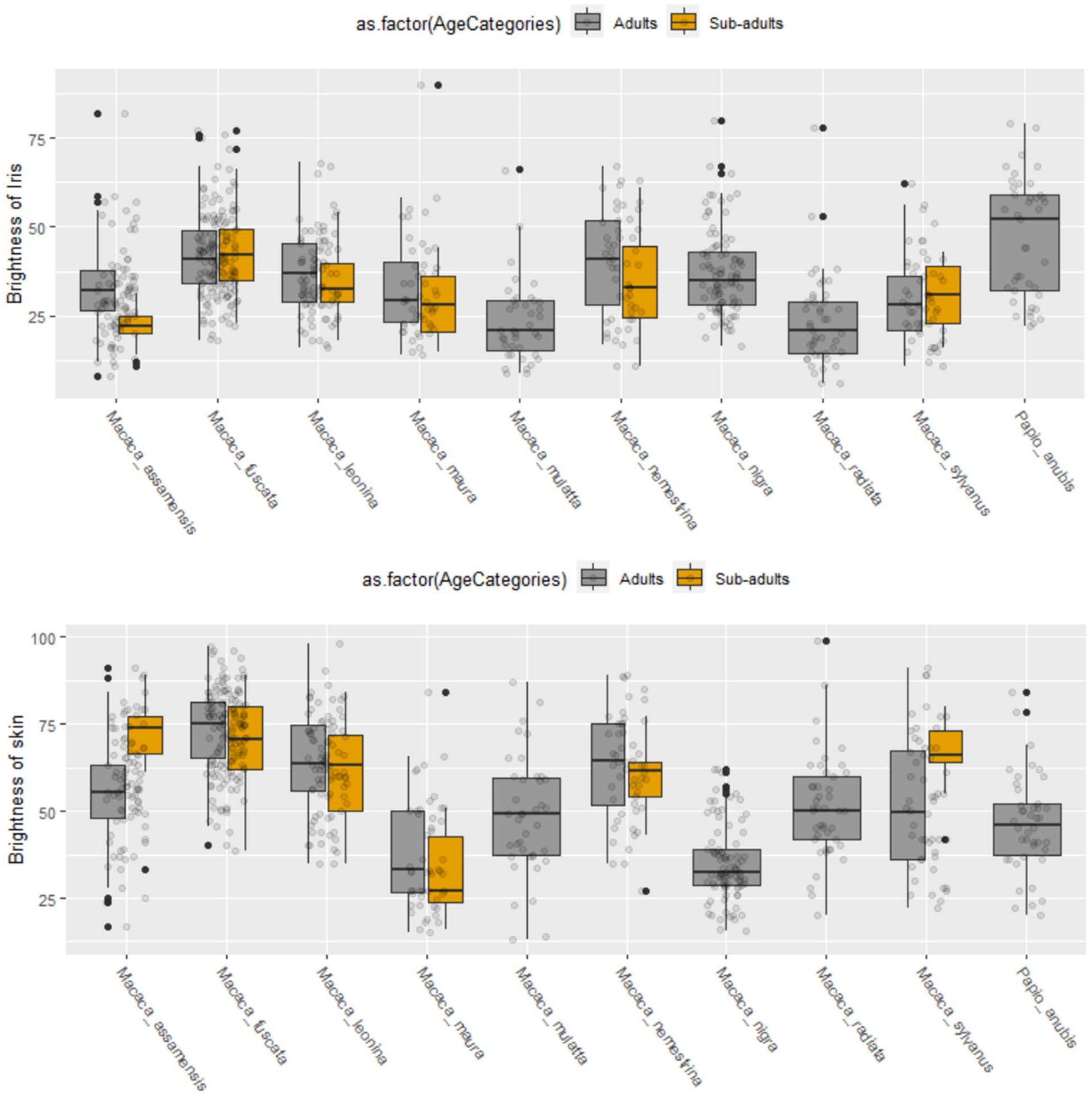

Figure S7.- Measurements of brightness of the APIT, PPIT, iris, and skin by species and age category. The figure shows raw measurements. The midline shows the median, and lower and upper hinges represent 25th and 75th percentiles, respectively. Black dots represent outliers.

There were differences in our measurements between sexes and age groups for each species in our sample. Measurements of brightness of the iris, APIT, PPIT, and skin brightness were generally higher in females, which is in line with preliminary evidence that pigmentation of the APIT is greater in adult males in orangutans [14]. However, the difference in our sample was generally very small. This makes us doubt whether these differences are of biological significance. Instead, the differences we found may be related to interactions between sex hormones and melanin synthesis - some evidence found decreased pigmentation levels in orchidectomised guinea pigs [54]. However, melasma was more prevalent in human males with

reduced testosterone levels compared to a control group in a recent study [55]. It is therefore unclear whether differences in androgens are enough to explain the differences we observed.

Differences between age groups were more consistent and greater than those between sexes. Measurements of brightness of the APIT and PPIT were higher in infants compared to adults, which is in line with evidence of a developmental component of ocular pigmentation over the course of development in chimpanzees [15]; [7] and bonobos [15]. Differences in measurements of iridal brightness were generally small and followed species-specific patterns. Differences in skin brightness also did not follow a clear age-related pattern across species, with small differences except for *M. assamensis* and *sylvanus* (15 brightness units). These sex and age differences call for more detailed examinations at the species level to determine their functions, if any, as well as the mechanisms underlying them.

Though there were differences in ocular measurements between sexes and age groups, these seemed species-specific and, in some cases, they appear to be so small that we doubt their biological significance. The largest and most consistent differences seem to be between age groups, perhaps indicating developmental constraints rather than functional differences. In general, the lighter parts of the eye in infants could increase detectability. Detectability by parents may benefit infants, but it is probably a trade-off with detectability by predators. As to photo-regulatory functions, it is unlikely that the reduced pigmentation observed in subadults is functional - at least in human infants, UV transmittance of the eyeball is several percent greater than in adulthood [48].

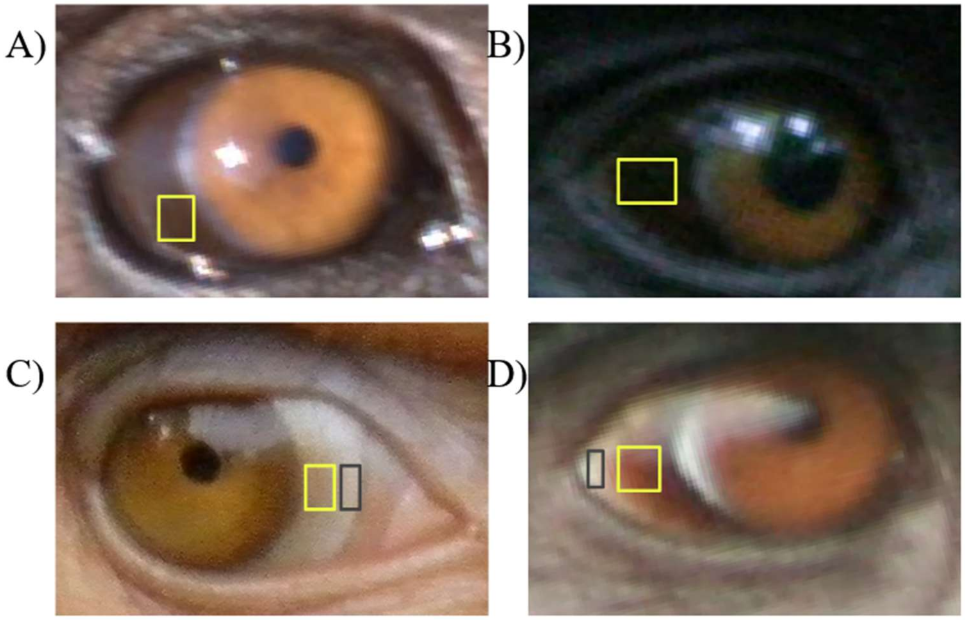

Figure S8.- Typical scenarios that illustrate our measurement process, focusing on distinguishing between “Anterior Peri-Iridal Tissues” (APIT) and “Posterior Peri-Iridal Tissues” (PPIT). Yellow squares were sampled as APIT; dark grey squares were sampled as PPIT. A)

No apparent difference in brightness in the peri-iridal tissues of an individual of *P. anubis*, which resulted in only taking measurements of APIT from this photograph. Spots of specular brightness distal to the iris and at the height of the pupil are also avoided; B) No clear distinction in brightness leading to only sampling as APIT in *M. nigra*; C) Faint but consistent ring of more pigmented tissue around the iris, sampled as APIT, and noticeably more depigmented tissue in the periphery, sampled as PPIT in *M. nemestrina*; D) Distinctly more pigmented tissue around the iris sampled as APIT, and noticeably more depigmented tissue in the periphery, sampled as PPIT in *M. nigra*.

Table S5.- Summary of results excluding samples from the population of *Papio anubis*

**Model**	**Latitude Affenberg Population**	**Intercept**	**β**	**SE**	**t**	**p**
Gaze-Camouflage: Iris and Surrounding Tissues, Social Grade	Population of Origin	3.0008	0.0566	0.2843743	0.1989694	0.8424
Gaze-Camouflage: Iris and Pupil, Social Grade	Population of Origin	6.1379	-0.4097	0.3451297	-1.187164	0.2357
Gaze-Camouflage: Iris and Surrounding Tissues, male-male coalitions	Population of Origin	2.5547	0.0125	0.2463407	0.0508288	0.9595
Gaze-Camouflage: Iris and Pupil, male-male coalitions	Population of Origin	21.8519	-2.5330	2.903752	-0.8723064	0.3835
Self-Domestication 1	Population of Origin	1.9200	-0.0586	0.08371935	-0.699739	0.4842
Self-Domestication 2	Population of Origin	1.7899	0.2516	0.1135395	2.216195	0.0269
Photo-Regulatory: Iris Blueness	Population of Origin	0.2477	-0.00003885	0.00107422	-0.036166	0.9712
Photo-Regulatory: Skin Brightness	Population of Origin	26.7710	1.1847	0.283224	4.182844	0.000

Photo-Regulatory: Anterior Peri-Iridal Brightness	Population of Origin	1.8747	-0.0001877	0.0038883	-0.048276	0.9615
Photo-Regulatory: Posterior Peri-Iridal Brightness	Population of Origin	4.8443	0.0857	0.0373724	2.292124	0.0225
Photo-Regulatory: Iris Blueness	Translocated	0.2185	0.0013	0.00059449	2.116421	0.0345
Photo-Regulatory: Skin Brightness	Translocated	44.2097	0.3966	0.156864	2.528189	0.0116
Photo-Regulatory: Anterior Peri-Iridal Brightness	Translocated	1.7598	0.0049	0.00237148	2.088173	0.037
Photo-Regulatory: Posterior Peri-Iridal Brightness	Translocated	4.5344	0.0983	0.0241126	4.075020	0.0001

Concerns regarding the adequacy of the method employed in this manuscript

Suitability of measurements from photographs across different studies

An earlier submission of this manuscript was rejected on the basis of methodological concerns expressed by a reviewer. This reviewer opined that non-standardized photographs cannot be used for meaningful measurements. The reviewer in question pointed at work by Whitham et al. [41], [42] as representative of “old [...], well-established [...], free and open source”. In doing so, they characterize the method we employ as “nonstandard”, and imply that the measurements are too susceptible to error from ambient lighting to be used effectively for the purposes of the study. The reviewer’s main critique is reproduced below in full:

Techniques for measuring animal coloration, are old (e.g., Endler, 1990), well-established (e.g., Vorobyev & Osorio, 1998, Proceedings of the Royal Society B: Biological Sciences), have been previously applied to primates (e.g., Allen et al., 2014, Nature Communications; Bergman & Beehner, 2008, Biological Journal of the Linnean Society), have been previously applied to primate eyes (e.g., Whitham et al., 2022, Scientific Reports), and are free and open-source to anyone who wants to use them (e.g., Stevens et al., 2007, Biological Journal of the Linnean Society; Troscianko & Stevens, 2015, Methods in Ecology and Evolution). The authors appear to report a nonstandard analysis instead of anything like the established tools for scientific color analyses presented above, a decision that probably cannot be justified.

Most of these concerns should be addressed by the methodological work by Laitly et al. [29]. Nonetheless, the reviewer misconstrued the contents of Laitly et al’s article in their review. More specifically, the reviewer writes that:

“Laitly et al., 2021 should not be taken to mean that at any 10-12 images, of any species, are sufficient for any question. That paper includes many cautions about the variability introduced by individual cameras, and functions to describe what can be learned (about bird coloration) by averaging across the parametric variability of digital images from various sources. Achromatic information is typically more difficult to measure, is most critical to the hypotheses of this manuscript, and is not well addressed by Laitly et al.’s comparisons of crowdsourced images vs museum specimen ground truth.”

Lastly, the reviewer expressed a series of concerns that may have resulted from a lack of attention to the manuscript itself. Mainly, the reviewer expressed concerns about whether age is not accounted for in our study, and whether we may be generalizing population values to species values:

Subject variables other than sex and species may matter as well. Were these animals living in captivity? In the wild? Young? Old?

Taken together: When the authors report that an eye region appears “more blue” or “more pigmented” is that a characterization of a species? Or of a particular camera? Camera settings?

The light environment of a particular enclosure of a particular species? Something about the demographics of the sample? Their ideas and hypotheses cannot be evaluated with the sparse information about how all data were collected, and from the limited information available it appears that data were collected in a way that deviates substantially from norms in the study of animal coloration.

These are not reasonable critiques of the method we employ. Firstly, the reviewer portrays a partial review of the literature that exaggerates the degree to which their proposed methods are standard. If one were to solely rely on the reviewer's paragraph, it would seem as if a very specific methodology were standard in animal coloration studies since the 1990s. This is not the case:

a. New methods do not necessarily supplant older methods, that may still provide adequate data, depending on the research question. The reviewer omitted that the inaugural study on primate eye coloration used entirely subjective scoring of photographs [1].
b. Indeed, variations of the "subjective" method continue to be used: by Zhang & Watanabe in 2007 [44], Mayhew & Gómez in 2015 [2], and again by Clark et al in 2023 [7]. The reviewer's proposed method has not supplanted subjective assessments. This extends to systems other than primates, such as amphibians [56]
c. Methods like ours, relying on digital quantification of a large number of uncalibrated photographs are pervasive and advance their respective fields (e.g. [57]; cf [58] for a review of diverse methods that continue to be used in assessing specifically bird eye coloration), particularly where they allow for the analysis of large diverse samples that better represent species-level characteristics
d. Only two studies employ the method proposed by the reviewer to assess ocular coloration in primates [41]; [42].

2. The reviewer implies that, because our method is more error-prone than their proposed method, it results in meaningless measurements. Below, we show graphs demonstrating that this is not the case, and that the method retains at least internal validity across studies by the same and different authors.
3. The reviewer does not entertain that variability due to source of lighting should even out, given that there is not a systematic bias in our samples. Our samples were taken in different conditions of lighting due to angle, time of the day, cameras, position of the animals, etc. The only constants are the subjects. This is the reasoning in Laitly et al.'s [29] both about achromatic and chromatic information. Both are underlied by the same assumptions, including the law of large numbers - as are all inferential statistics methods.
4. The reviewer's comment about achromatic information is puzzling, as it contradicts Laitly et al.'s [29] conclusions. It is true that the focus of their study is chromatic information, but they also explicitly assess the reliability of achromatic information from unstandardized photographs. Several excerpts and data from the article directly contradict the reviewer's conclusion that achromatic measurements are more error

prone with our method, and that Laitly et al.'s [29] results cannot be used to support our use of achromatic (brightness) measurements, among which these stand out:

   a. *"In our analysis of how well color in C[itizen] S[cience] photographs corresponds with measurements in bird visual space, we found a strong relationship between the achromatic components from each space, with an R^2 value of 0.73 (Table 2)."*
   b. In Table 1, achromatic (brightness) measurements from uncalibrated photos using the same color space as our study (HS**V**) obtain 0.71 R^2 with measurements from museum specimens.

5. The reviewer's statement that the methods they propose are "*and are free and open-source to anyone who wants to use them*" is technically true, but disingenuously misleading for the present case. As explained above, their proposed method requires photographs to be taken specifically for these purposes. It would be simply logistically unfeasible to include the number of species, individuals, and sites that we include using their proposed method - and that are essential to properly describe the species-level differences we are interested in.
6. The two papers that use the method the reviewer referred to include only one site per species, which could lead to the reviewer's own criticism when they ask whether we are measuring a population, or a species. This is generally a limitation that is accepted when characterizing species from a limited set of animals.
7. The reviewer does not seem aware that we have segregated measurements according to age, in addition to sex and species.
8. The reviewer implies that ocular coloration may change depending on whether the population is captive or wild. If the reviewer refers to literature showing that this is a genuine concern, we will be happy to provide these data. For species-level characterizations, we believe our usage of wild/captive populations is irrelevant - just like in the two papers where the authors use the method recommended by the reviewer.

Comparability of species-level measurements across different studies using the method we employ in the manuscript

Below, we display a side-by-side comparison of grayscale values of the sclera and iris taken in different studies using the same method as we employ here. The overlap is more substantial than one would expect if our measurements simply represented "noise", especially in the species of interest for our current manuscript (*Macaca spp.*).

The species in which there is considerable divergence across studies, are species in which there is actually a very variable external eye appearance at the species level - most notably, the great apes. This pattern is already reported in Mayhew & Gómez [2] with regards to gorillas; [14] with regards to orangutans, [5] with regards to hominoids, and [7] with regards to chimpanzees. Thus, results from studies using the same method are providing replicable, comparable findings.

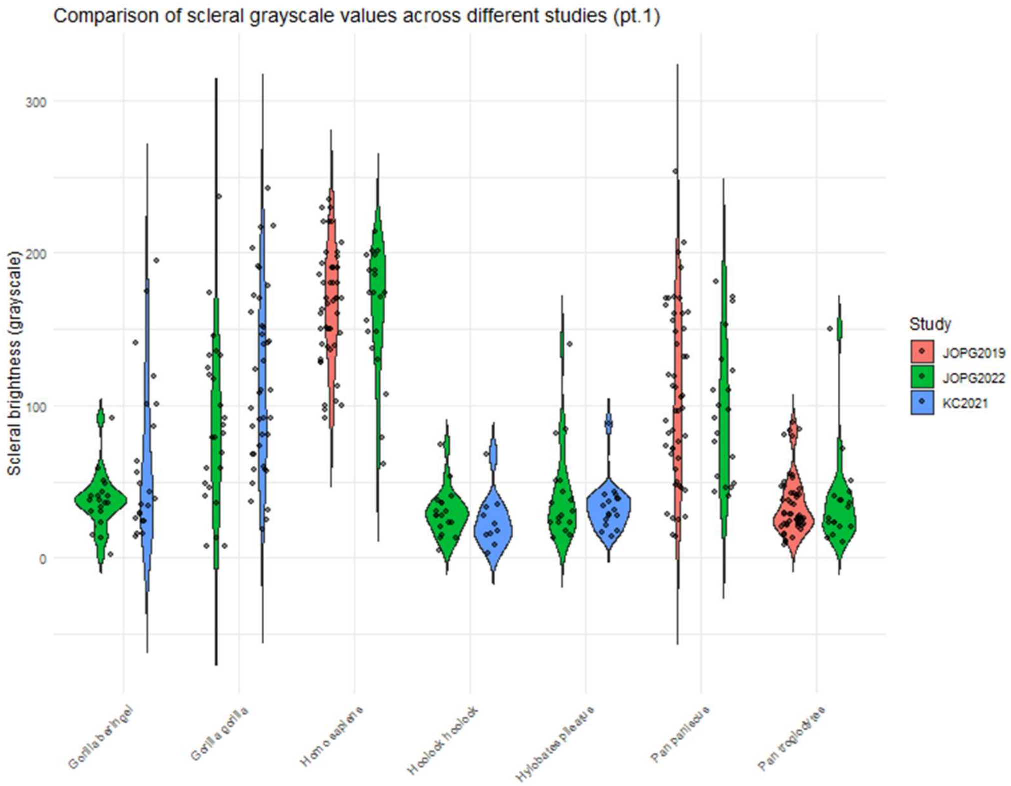

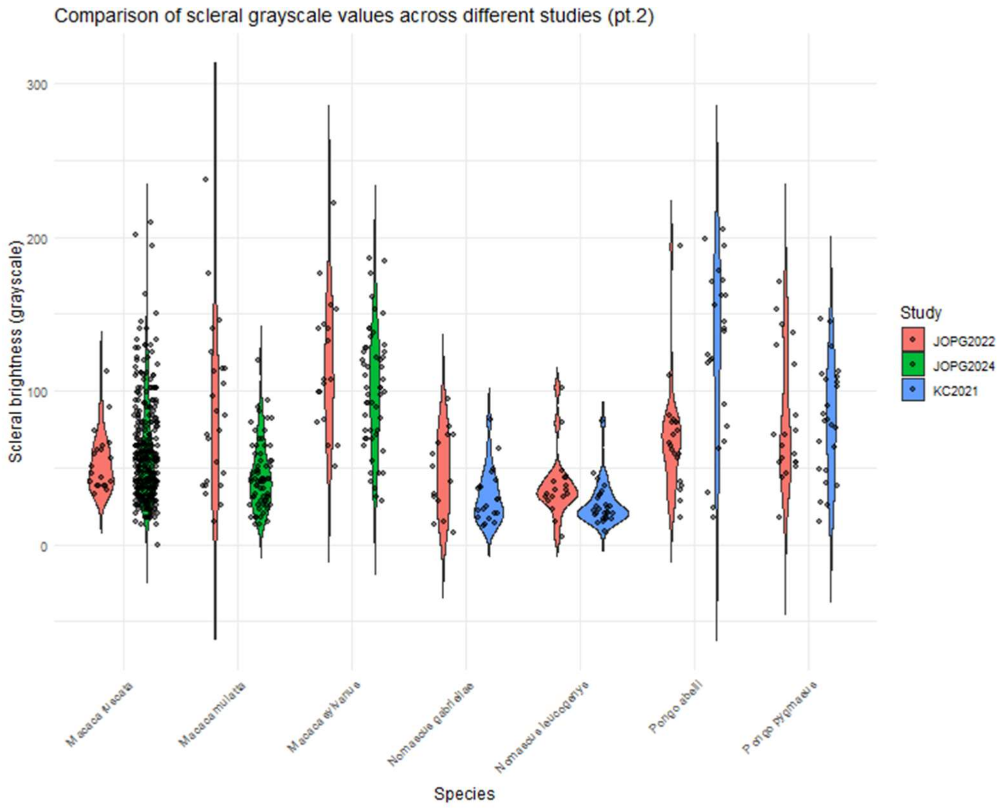

Figure S9.- Comparison of scleral grayscale values across different studies

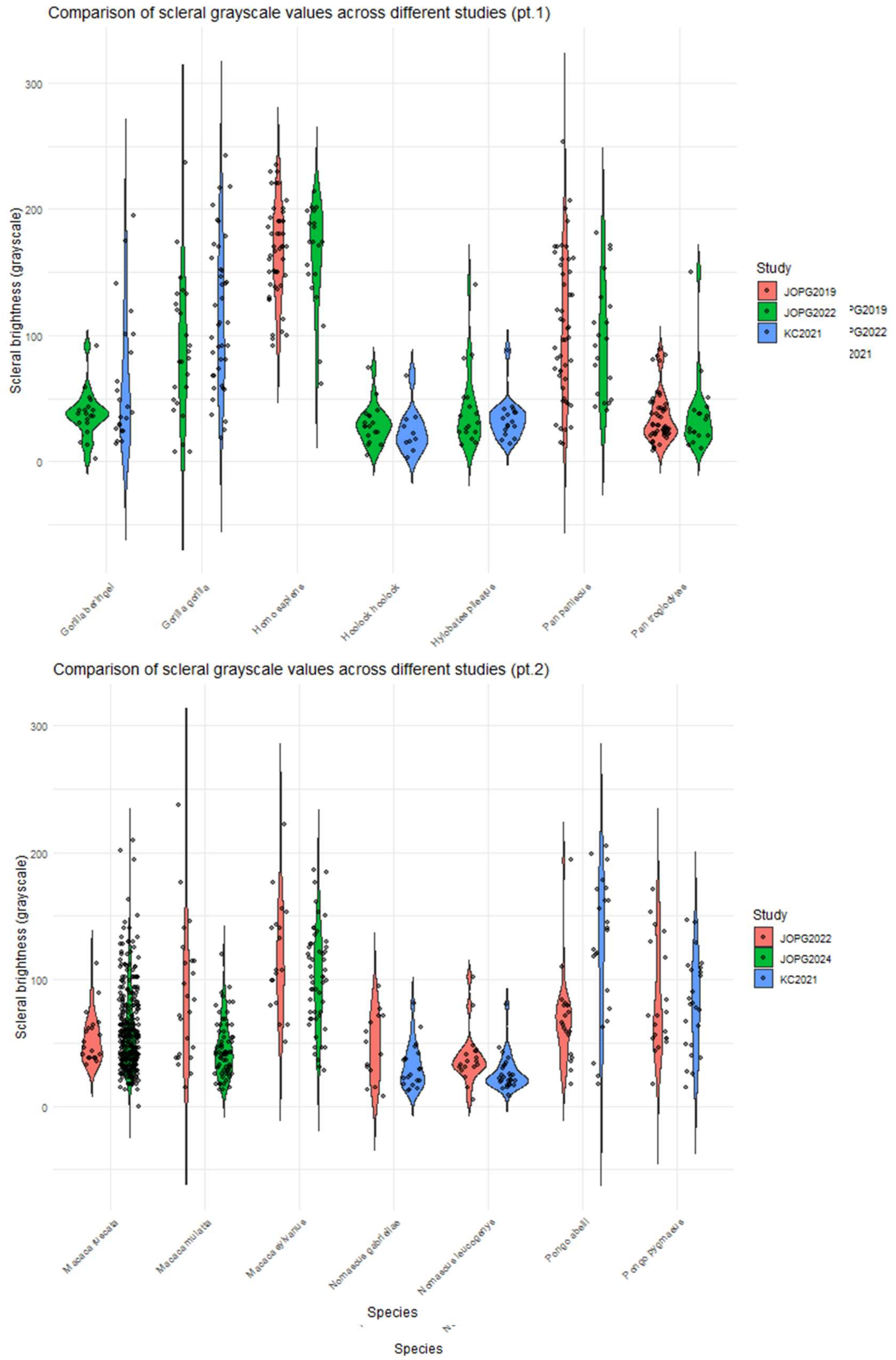

Figure S10.- Comparison of iridal grayscale values across different studies

Suitability of the method for comparison across studies using different methods

The graphs above show that species-level measurements are comparable across studies using variations of the method we use (e.g. large number of measurements from uncalibrated photographs). With attention to methodological differences, comparison across studies also seems feasible.

Namely, both studies using the method proposed by the reviewer, arrive at similar conclusions as studies using the method we employ here. Most notably (emphasis our own):

Whitham, W., Schapiro, S. J., Troscianko, J., & Yorzinski, J. L. (2022a). **Chimpanzee** (Pan troglodytes) **gaze is conspicuous** at ecologically-relevant distances. Scientific reports, 12(1), 9249.

Perea-García, J. O., Kret, M. E., Monteiro, A., & Hobaiter, C. (2019). Scleral pigmentation leads to **conspicuous**, not cryptic, **eye morphology in chimpanzees**. Proceedings of the National Academy of Sciences, 116(39), 19248-19250.

Lastly, measurements in *Sapajus* (*Cebus*) *apella* using our method (in [11]) placed the species above the average of measurements of contrast, suggesting that it is not cryptic and may be conspicuous (contra earlier studies like [1]). This same conclusion was also reached when using the reviewer's proposed method [41]:

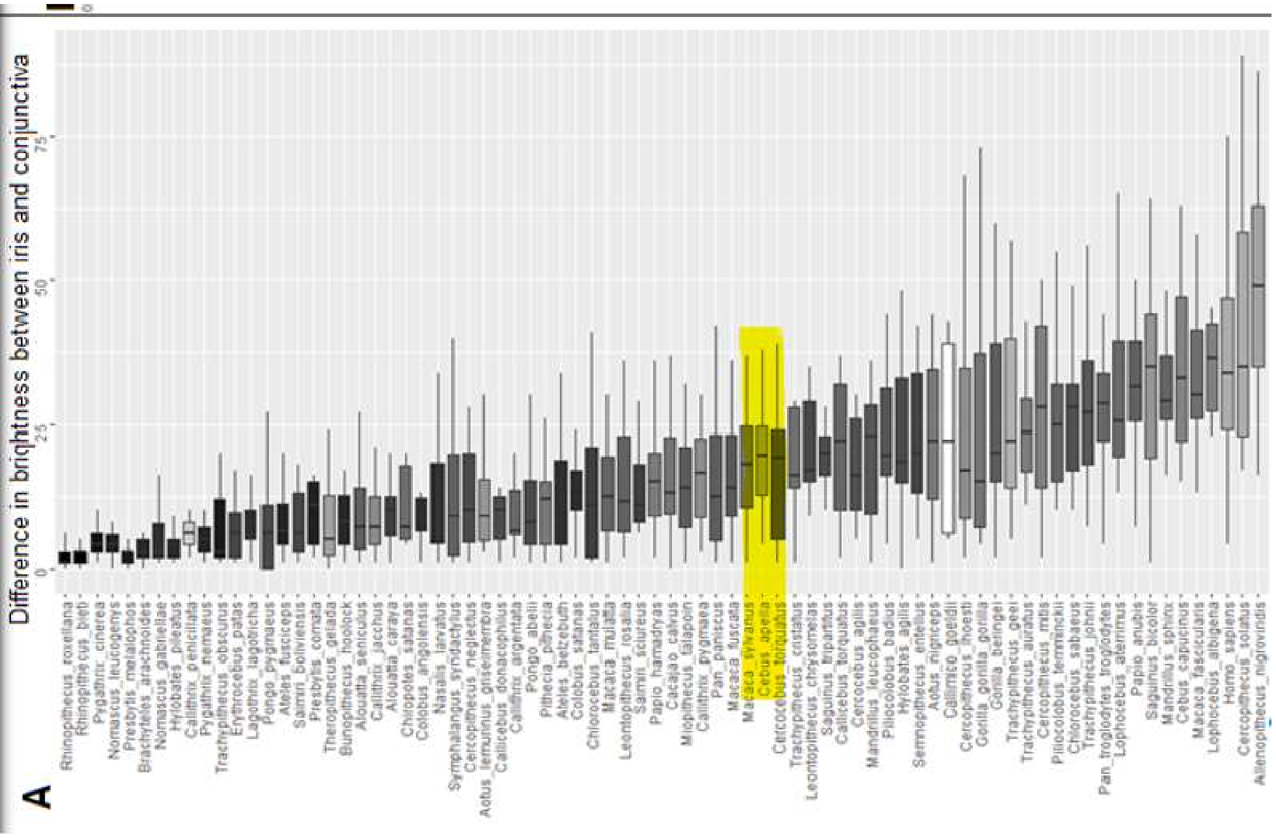

Supplement: Supplementary file 1 — Supplementary Material 1 [file 41598_2024_80643_MOESM1_ESM.pdf]
